# PROTACs: The Future of Leukemia Therapeutics

**DOI:** 10.3389/fcell.2022.851087

**Published:** 2022-09-02

**Authors:** Zubair Anwar, Muhammad Shahzad Ali, Antonio Galvano, Alessandro Perez, Maria La Mantia, Ihtisham Bukhari, Bartlomiej Swiatczak

**Affiliations:** ^1^ Department of Surgical, Oncological, and Oral Sciences, Section of Medical Oncology, Uiniversity of Palermo, Palermo, Italy; ^2^ Department of Clinical and Biological Sciences, University of Turin, San Luigi Hospital, Turin, Italy; ^3^ The Fifth Affiliated Hospital of Zhengzhou University, Zhengzhou, China; ^4^ Department of History of Science and Scientific Archeology, University of Science and Technology of China, Hefei, China

**Keywords:** PROTACs, anticancer therapeutics, leukemia, cancer, linker

## Abstract

The fight to find effective, long-lasting treatments for cancer has led many researchers to consider protein degrading entities. Recent developments in PROteolysis TArgeting Chimeras (PROTACs) have signified their potential as possible cancer therapies. PROTACs are small molecule, protein degraders that function by hijacking the built-in Ubiquitin-Proteasome pathway. This review mainly focuses on the general design and functioning of PROTACs as well as current advancements in the development of PROTACs as anticancer therapies. Particular emphasis is given to PROTACs designed against various types of Leukemia/Blood malignancies.

## Introduction

In the last few decades, numerous advancements have been made in developing protein-degrading complexes as treatments against a wide range of diseases. Perhaps the most exciting of these are PROTACs or Proteolysis Targeting Chimeras. PROTACs are bivalent, heterobifunctional small molecules that prompt the destruction of the Protein of Interest (POI) (Maniaci et al., 2017; X.; Sun et al., 2019). They are composed of two ligands, one for the POI (Protein of Interest) and the other for an E3 ubiquitin ligase that are coupled together by a linker. PROTACs utilize the inherent Ubiquitin-Proteasome Pathway of the cell. They stimulate the E3 ligases and POI to form a complex, thus triggering the ubiquitination and, ultimately, the degradation of the Protein of interest by 26S Proteasome (Sakamoto, 2010; Weng et al., 2021).

One of the most eminent uses of PROTACs has been their development for cancer therapeutics. According to GLOBOCAN 2020, cancer is amongst the leading causes of global mortality, with the rate of incidence and mortality increasing rapidly. In 2020, approximately 19.3 million new cases of cancer were recorded, along with 10 million deaths (Sung et al., 2021). Of these, nearly 2.5% new cases and 3.1% deaths are due to leukemia (Sung et al., 2021). The burden of cancer has been estimated to rise by 47% in 2040, if the rate of new cases remains the same as that in 2020 (Sung et al., 2021).

Compared to other currently available antitumor treatments, PROTACs show a greater capacity for beneficial results (He et al., 2020). Conventional therapeutics for cancer treatment involve chemotherapy using cytotoxic drugs. These may prevent cell proliferation but do not specifically target cancer initiating cells/cancer stem cells and can thus provoke detrimental consequences (X. Li & Song, 2020). Advancing therapies, other than PROTACs, include nucleic acid based therapies, monoclonal antibodies and small molecule inhibitors. Nucleic acid based therapies, such as CRISPR, target DNA or RNA molecules and nucleotide analogues, to control the expression of proteins. However, such technologies have limitations including restricted tissue penetration and unavailability of oral admission (He et al., 2020; X.; Li & Song, 2020; X.; Sun et al., 2019). Similarly, monoclonal antibodies also lack oral bioavailability and can only target membrane proteins (He et al., 2020; J.; Liu et al., 2020; X.; Sun et al., 2019). Small molecule inhibitors were a promising breakthrough, with many being currently used as cancer treatments. Nonetheless, certain unavoidable challenges against them have risen, including drug resistance as a result of mutations, inability to target “undruggable” proteins and high dose requirements due to lack of catalytic mode of action (An & Fu, 2018; He et al., 2020; X.; Li & Song, 2020; J.; Liu et al., 2020; X.; Sun et al., 2019). PROTACs provide an opportunity to develop an approach that can overcome most of the shortcomings of previously available treatments; particularly drug resistance, targeting “undruggable” (due to absence of active site) proteins and debilitating protein functions that are nonenzymatic by degrading the whole molecule (An & Fu, 2018; J.; Liu et al., 2020).

PROTACs were first developed in 2001 (Sakamoto et al., 2001), against Methionine aminopeptidase-2, by (Khan et al., 2020; X.; Zhou et al., 2020). By 2003, PROTACs as a potential treatment for breast and prostate cancer had been created (Khan et al., 2020). Subsequently, the past 20 years has seen much advancement in PROTACs technology as well as progress in their use as possible anticancer therapies. Currently, the PROTACs ARV-471 and ARV-110 are involved in clinical trials for their use as estrogen receptor (breast cancer) degraders and androgen receptor (prostate cancer) degraders, respectively (Neklesa et al., 2018; Flanagan et al., 2019). They have shown promising results in phase I clinical trials (Qi et al., 2021).

The focus of this review will be on PROTACs designed as a potential anticancer treatment, particularly against leukemia. Leukemia is characterized as an assortment of hematopoietic (blood and/or bone marrow) disorders (Miranda-Filho et al., 2018; Dong et al., 2020). Leukemia incidence and mortality rates have increased from 437,033 to 309,006, respectively, in 2018 (Bray et al., 2018) to 474, 519 and 311, 594, respectively, in 2020 (Sung et al., 2021). Small molecule inhibitors (particularly tyrosine kinase inhibitors) proved a great breakthrough in increasing survival rates amongst Leukemia patients (Burslem et al., 2019). However, drug resistance, life-long use and chances of relapse pose serious concerns (Mahon et al., 2010; Corbin et al., 2011; Cao et al., 2021).

Numerous PROTACs are being developed against oncoproteins considered crucial in the development of various types of leukemia, particularly against the BCR-ABL, CDK, BTK and BET family proteins (Bond et al., 2020; Brand et al., 2019; Buhimschi et al., 2018; Dominici et al., 2020; Gadd et al., 2017; H.-T.; Huang et al., 2018; Jaime-Figueroa et al., 2020; Lai et al., 2015; Ward et al., 2019; Zhao et al., 2019; H.; Zhou et al., 2019).

## PROTACS: Basic Design and Functioning

### PROTACs Form Ternary Complexes

PROTAC employs a warhead, a linker and an E3 ligase binding moiety to promote targeted proteolysis by hijacking the inherent ubiquitin-proteasome pathway of the cells. Research has shown that the PROTACs form a ternary complex with the POI and E3 ligase by being positioned between them, a concept that was first visualized in 2017 (Gadd et al., 2017). Gadd et al. (2017) used X-ray crystallography to confirm the formation of ternary structures between MZ1 (PROTAC), Brd4 (BET family proteins) and VHL (Von Hippel–Lindau protein). Two asymmetrical ternary complexes, with minor deviations, were observed in the following orientation: Brd4-MZ1-VHL (Gadd et al., 2017). This orientation allowed formation of novel interactions between the proteins, as well as between the proteins and their ligands, resulting in a more stable structure as well as specific folding of ligands to allow improved recruitment and productivity of targets (Gadd et al., 2017).

### PROTACs Hijack the Intrinsic Ubiquitin-Proteasome Pathway

**FIGURE 1:**
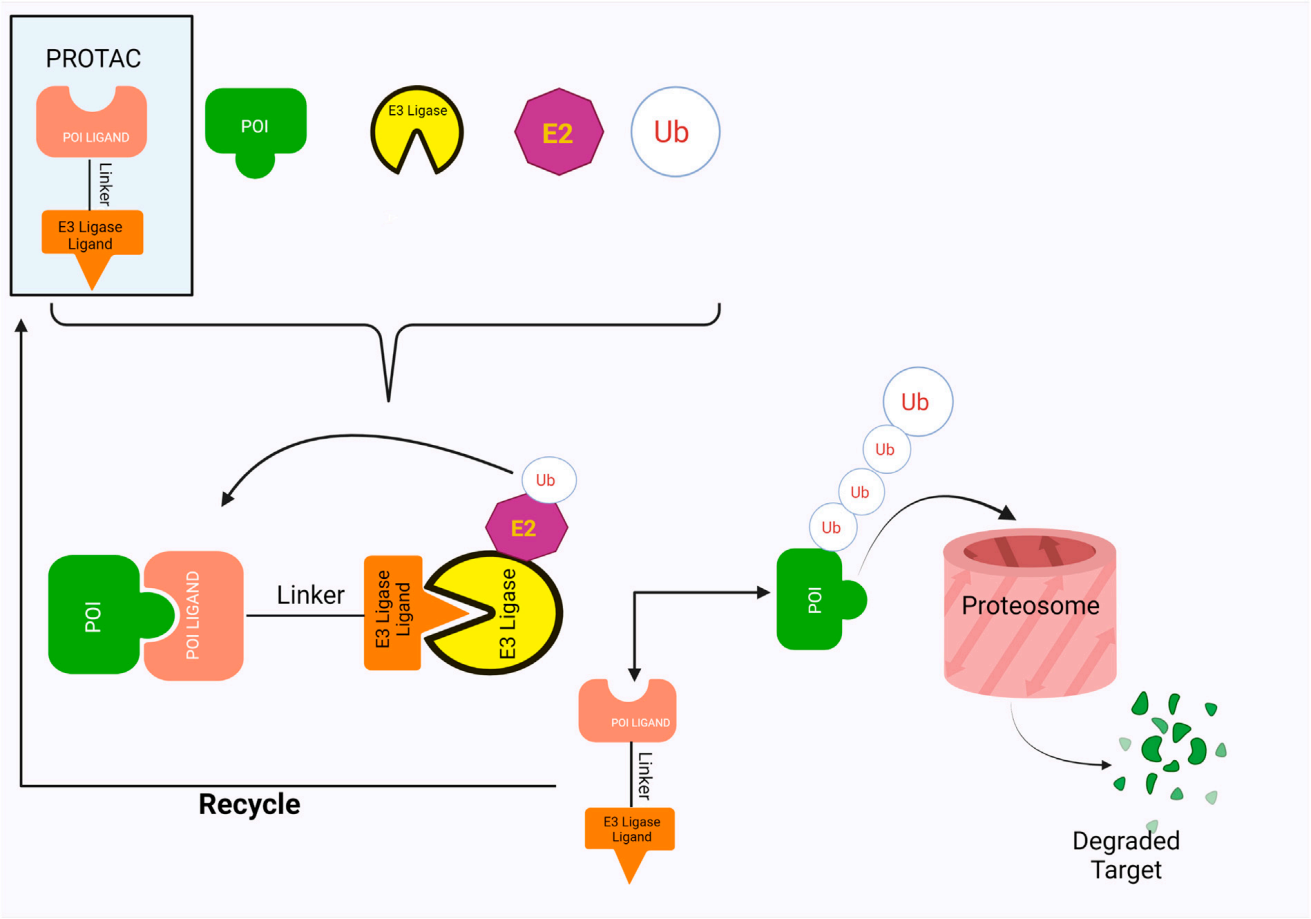
The PROTACs mode of action, involving ubiquitination and eventual degradation of POI.

The ligands in the PROTACs bind to their respective targets, POI and E3 ligases, thereby bringing them into close proximity of each other (Figure 1). This results in the transfer of ubiquitin from E2 ligase onto the POI, a process which is catalyzed by the E3 ligases (Yin & Hu, 2020). Successful ubiquitination of the POI marks it for destruction, which is thus accomplished by the proteolytic activity of the 26S proteasome (Yin & Hu, 2020). Furthermore, PROTACs employ a catalytic mode of action wherein they disconnect from complex after ubiquitination, thus showing great potential for exhibiting significant activity at low doses (X. Sun et al., 2019). This also allows them to be less vulnerable towards surges in target expression and/or mutations (X. Sun et al., 2019).

Initially, emphasis was placed on selecting ligands possessing high affinity for their respective substrates, however, further research has found that greater prominence needs to be given to the association between the specific ligase and the POI chosen (Bondeson et al., 2018). The novel Protein-Protein Interactions (PPI) between them result in a more stable ternary complex that produces a more successful PROTACs, as noted in several studies (Lai et al., 2015; Chan et al., 2018; Smith et al., 2019).

### Protein of Interests and Warheads

According to the PROTACs database, PROTAC-DB (http://cadd.zju.edu.cn/protacdb/), over a hundred proteins have been targeted for degradation by PROTACs. Amongst the most popular include nuclear receptors, kinases, epigenetic proteins (such as BET proteins and histone deacetylases), STAT3 and even E3 ligases itself (Hu et al., 2020; X.; Zhou et al., 2020). Concurrently, the database also reveals 275 warheads that have up till now been employed in PROTACs. Currently, majority of them are established warheads i.e. actively inhibiting proteins by binding to their respective active sites (Yin & Hu, 2020). Only a handful of PROTACs have used warheads that bind allosterically to their respective target proteins (Burslem et al., 2019). Research has shown that warheads with limited or non-existent inhibiting activity can still produce significantly positive results (Paiva & Crews, 2019).

### E3 Ligase Ligands

Furthermore, while more than 600 E3 ubiquitin ligases have been estimated to exist in the human genome, only a few of them have been used by PROTACs (Yin & Hu, 2020; Bricelj et al., 2021). The most popular of these are von Hippel-Lindau (VHL) and Cereblon (CRBN). VHL, together with Rbx-1, cullin 2, elongin B and elongin C, is a component of the CRL2VHL complex, a Cullin RING ligases (Smith et al., 2019; Bricelj et al., 2021). One of its two domains, which form after folding, behaves as a substrate binder, most particularly with HIF-1α (Hypoxia-inducible factor 1-alpha) (Smith et al., 2019). Crystal structures of VHL have proven their ability to bind with small molecule inhibitors in a method analogous to their binding with HIF-1α (Chen & Jin, 2020). This has made them ideal for use in PROTACs, one of the most popular being VH298 (Chen & Jin, 2020). Similarly, CRBN are a part of the Cullin-4-RING E3 ubiquitin ligase complex, where they act as specific substrate adapters (Bondeson et al., 2018; Bricelj et al., 2021). Generally, substrates for CRBN are immunomodulatory imide drugs or IMiDs (Bricelj et al., 2021), especially glutarimide compounds including thalidomide, pomalidomide and lenalidomide (Yin & Hu, 2020). Binding of the IMiDs to the CRBN results in modifications to their ligase activity, resulting in subsequent ubiquitination and proteolysis (Bricelj et al., 2021). Interestingly, E3 ligases c-Cbl (Casitas B-lineage lymphoma), C-terminus of Hsc70-interacting protein (CHIP) and chimeric ubiquitin ligase, SH2-U-box are involved to induce degradation of BCR-ABL (Tsukahara & Maru, 2010; Ru et al., 2016).

### Linkers

Although comparatively lesser importance has been given to linkers in the past, the significance of their composition and length on the stability and formation of the ternary complex, along with the proteolysis activity and target specificity, cannot be denied (Troup et al., 2020). However, as more focus has generally been on the warheads and E3 ligands selected, linkers have been optimized according to each individual PROTAC (Troup et al., 2020). Thus, there are no standard methods or rules for constructing linkers. Traditionally, linkers have been formed as a combination of several chemical motifs, particularly PEG and Alkyl motifs (Troup et al., 2020). According to PROTAC-DB, more than a thousand linkers have been developed to date.

## PROTACs Developed Against Leukemia Oncoproteins

### BCR-ABL Oncoproteins

The BCR-ABL oncoprotein is the product of reciprocal chromosomal translocation between the long arms of chromosome 9 and 22 result in the genesis of Chronic Myeloid Leukemia (CML) (Bracco et al., 2021). Its oncogenic properties are a result of its tyrosine kinase activity (Bracco et al., 2021). Generally, BCR-ABL + CML is treated with tyrosine kinase inhibitors (TKIs), usually requiring lifelong administration (T.-T. Huang et al., 2021). Several shortcomings have been associated with TKIs: First generation TKIs (Imatinib) show drug resistance and can result in oncoprotein overexpression; second generation TKIs (dasatinib, nilotinib, and bosutinib) show resistance against T3151 mutation; and third generation TKIs (Ponatinib) result in adverse cardiovascular side effects (T.-T. Huang et al., 2021; Pophali and Patnaik, 2016).

PROTACs against BCR-ABL were first developed in 2016, by linking imatinib, bosutinib or dasatinib to VHL or CRBN by any of 4 different linkers (Lai et al., 2015). Their activity was tested against K562 CML cells (Lai et al., 2015). Imatinib, although it bound to both ABL and BCR-ABL, showed no degradation (Lai et al., 2015). The boustinib-VHL PROTAC showed no degradation while the dasatinib-VHL PROTAC degraded ABL only (Lai et al., 2015). Both boustinib and dasatinib, when bound to CBRN, showed degradation of ABL and BCR-ABL (Lai et al., 2015). In the following 2 years, protein degraders using ABL kinase inhibitors and IAP ligands were used against BCR-ABL. SNIPER(ABL)2 (Table 2), imatinib connected to methyl bestatin by hexyl linker (Demizua et al., 2016), and SNIPER(ABL)-39 (Table 2), dasatinib and LCL161 derivative connected by polyethylene glycol (PEG) × 3 linker (Shibata et al., 2017), showed promising results. Consequently, researchers turned their attention towards allosteric sites on BCR-ABL as possible PROTACs targets. Shimokawa et al. were amongst the first to target allosteric site, thus developing SNIPER(ABL)-21 (Table 2), GNF-2/-5 (1–2) with LCL-161, and SNIPER(ABL)-62 (Table 2), ABL001 and LCL-161 (Shimokawa et al., 2017). Allosteric site targeting PROTAC GMB-475 (Table 2), developed using GNF-5 AND VHL, showed great potential, particularly against mutations that are clinically important (Burslem et al., 2019). The researchers also demonstrated the benefit of using protein degraders in unison with protein kinase inhibitors like imatinib (Burslem et al., 2019). The PROTAC that was established by linking dasatinib with VHL1, SIAIS178 (Table 2), also showed remarkable degradation and tumor regression (in K526 xenografts) capacity (Zhao et al., 2019). Furthermore, it degraded BCR-ABL containing clinically important mutations, which confer drug resistance, and, after treatment for a short period, exhibited a comparatively prolonged cellular response than inhibitors (Zhao et al., 2019). Other similar studies have been conducted against BCR-ABL proteins containing clinically important mutations, including one using the PROTAC SIAIS056, containing a sulfur-substituted carbon chain linker (H. Liu et al., 2021; Yang et al., 2020). A novel study involves the use of nimbolide, derived from Azadirachta indica, as a ligand for the E3 ligase RNF114, in combination with dasatinib to generate BT1 (Tong et al., 2020). Unlike previous studies involving VHL and CRBN, nimbolide showed greater degradation of BCR-ABL as compared to c-ABL (Tong et al., 2020).

**TABLE 1:** Comparison of small molecule inhibitors, monoclonal antibodies, nucleic acid based therapies and PROTACs as potential antitumor therapies.

	Small molecule inhibitors	Monoclonal antibodies	Nucleic acid based therapies	PROTACs
Target	Intracellular and cell surface proteins	Cell surface proteins only	DNA or RNA	Intracellular and cell surface proteins
Tissue Penetration	Broad	Limited	Limited	Broad
Oral bioavailability	Easily achievable	Not achievable	Not achievable	Achievable
Target Undruggable Proteins	No	Only membrane proteins	Not applicable	Yes
Target Scaffolding Proteins	No	Not Applicable	Not Applicable	Yes
Drug resistance due to mutations	Yes	Yes	Not applicable	No
Possibility for high drug potency	Poor	Yes	Yes	Yes
Mode of action catalytic	No	No	Yes	Yes

**TABLE 2:** PROTACs designed against BCR-ABL [Structures taken from PROTAC-DB (http://cadd.zju.edu.cn/protacdb/)]

PROTAC	Warhead	Linker	E3 ligand
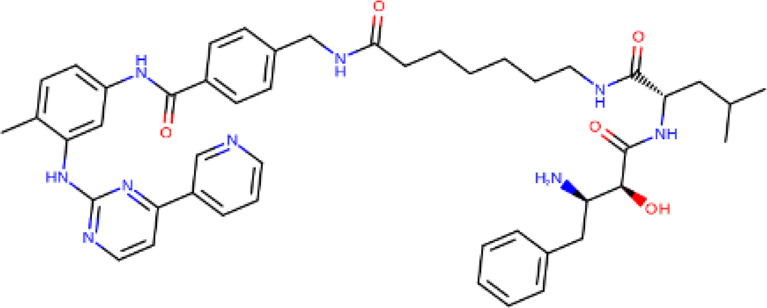 SNIPER(ABL)-2	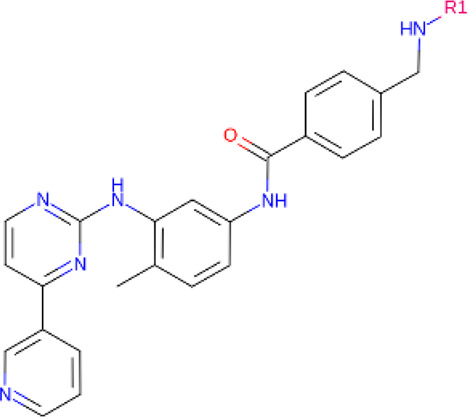	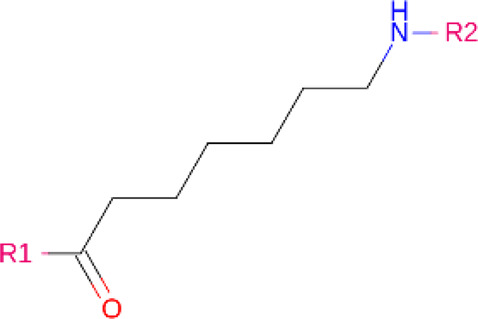	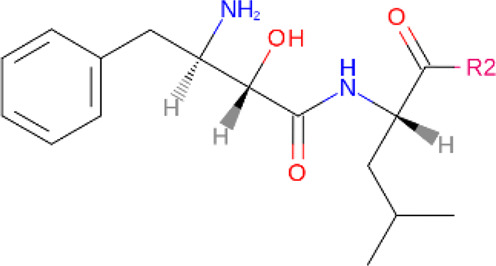 E3 ligase: cIAP1
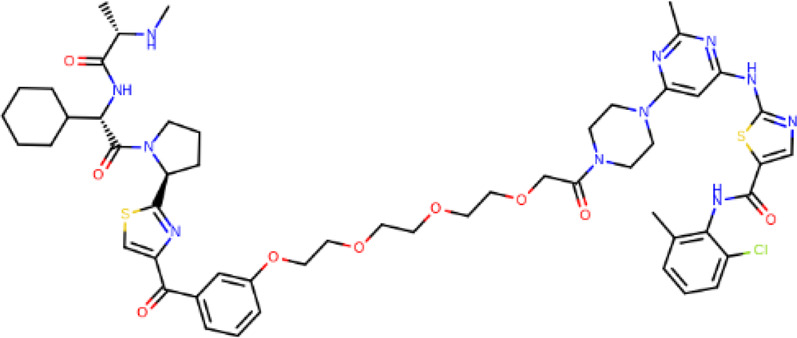 SNIPER(ABL)-39	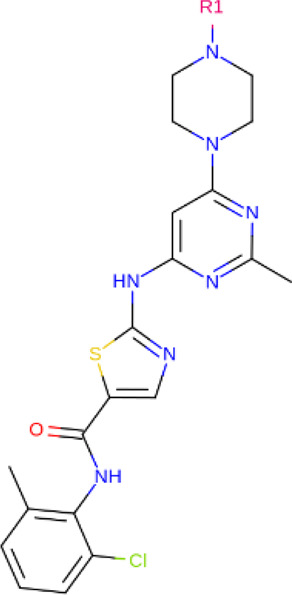	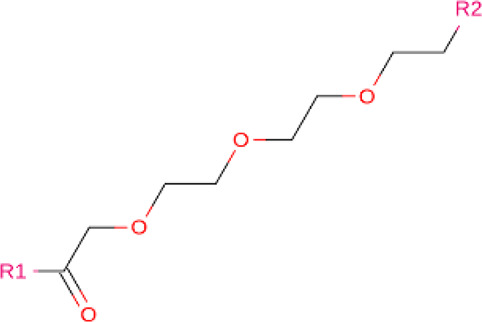	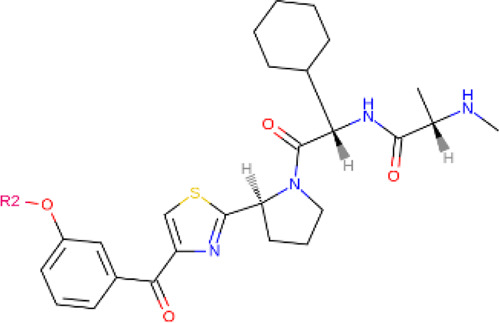 E3 ligase: cIAP1
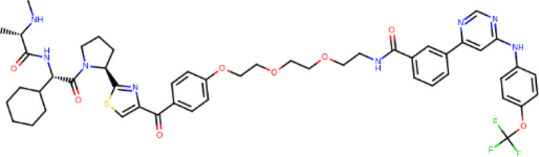 SNIPER(ABL)-21	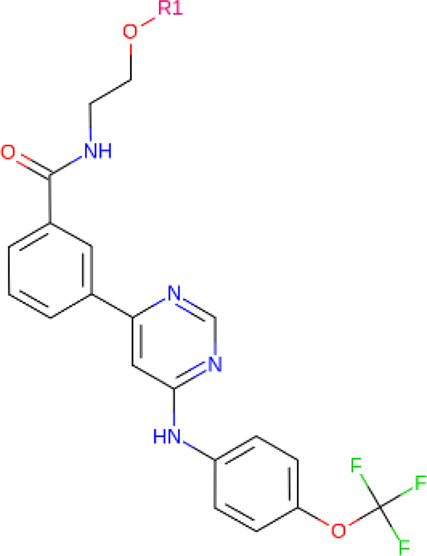	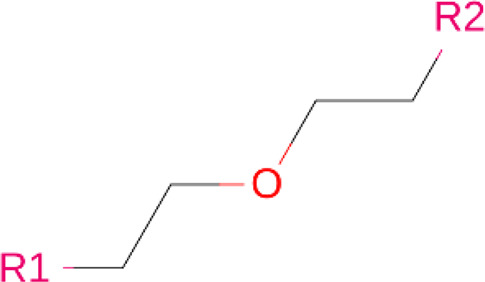	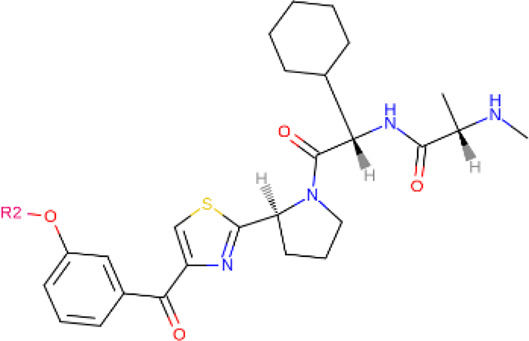 E3 ligase: cIAP1
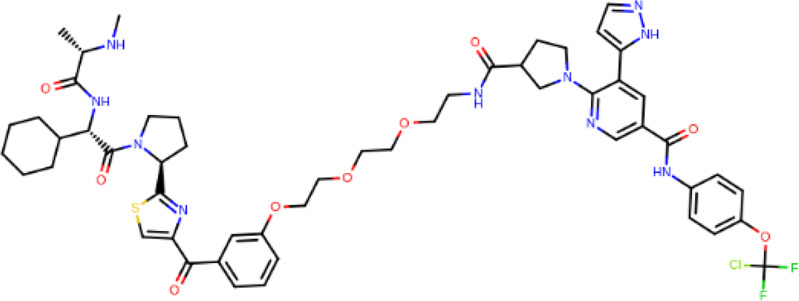 SNIPER(ABL)-62	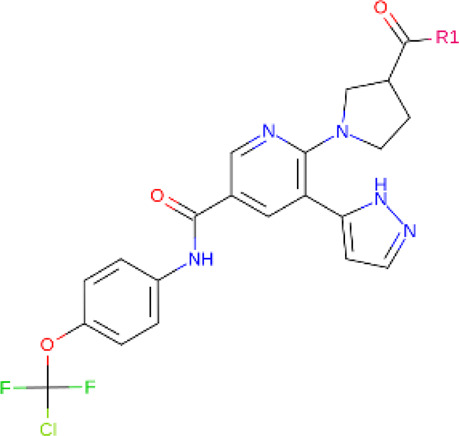	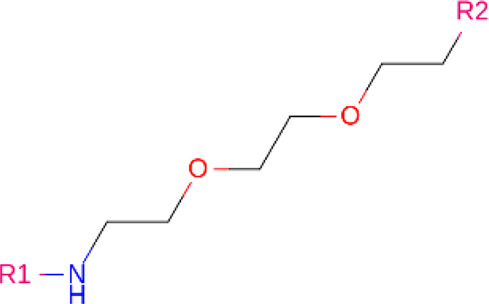	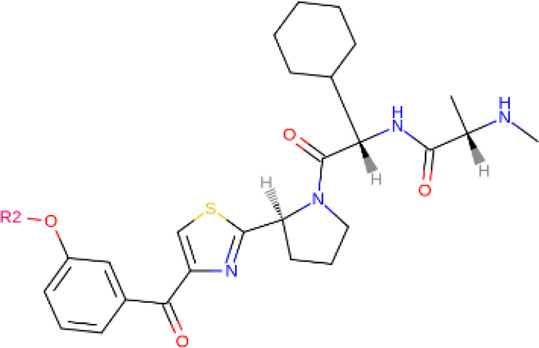 E3 ligase: cIAP1
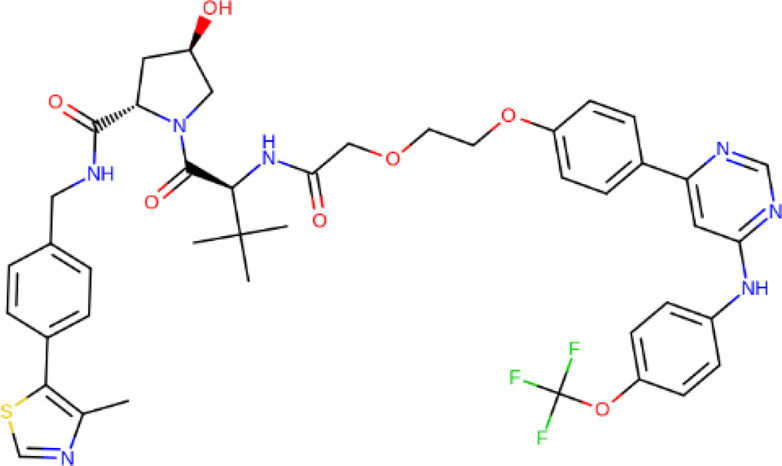 GMB-475	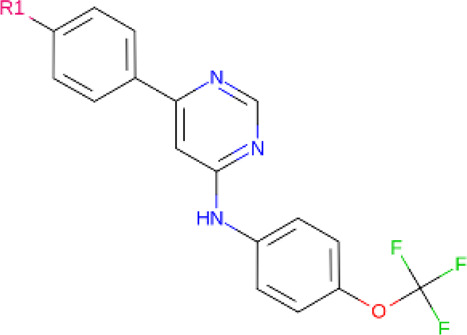	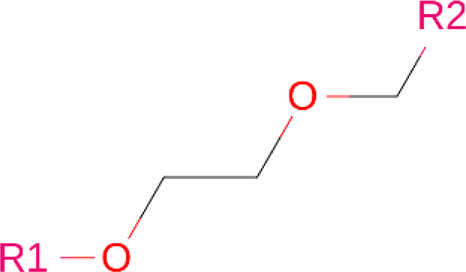	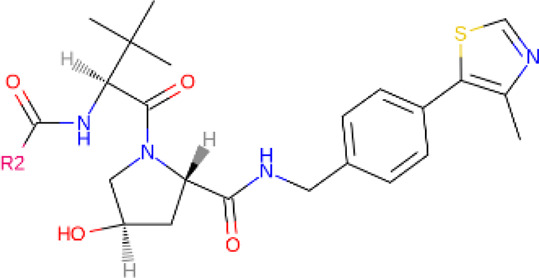 E3 ligase: VHL
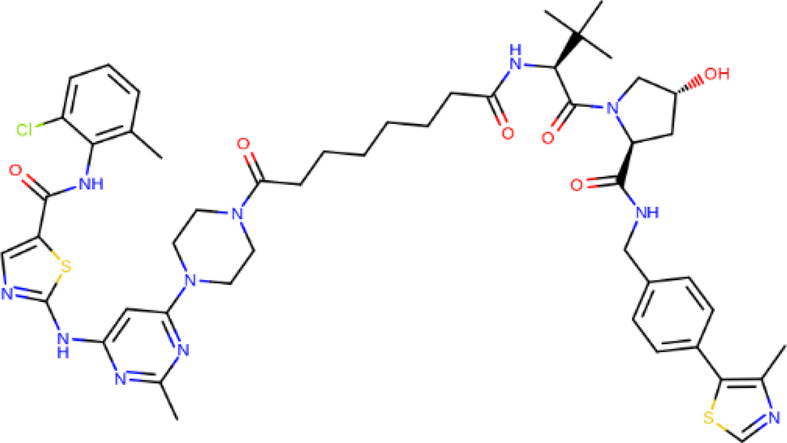 SIAIS178	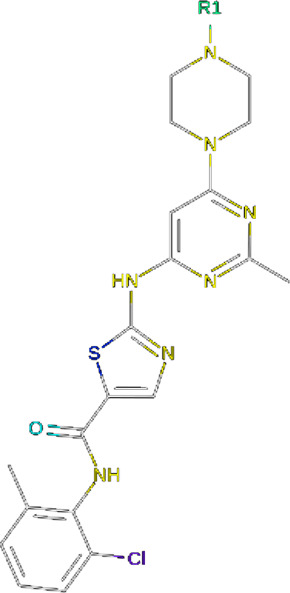	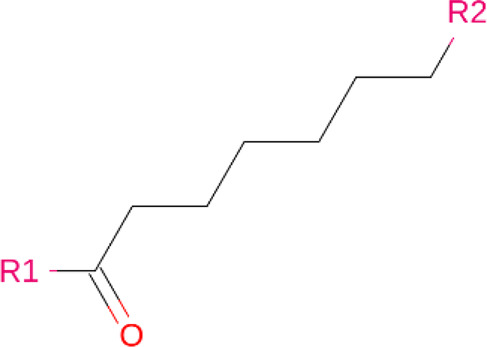	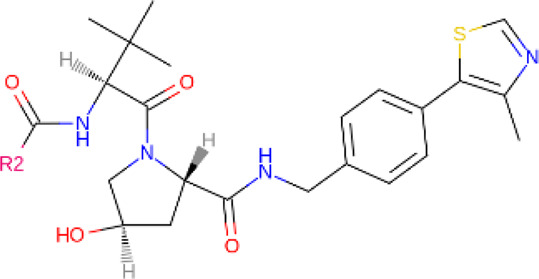 E3 ligase: VHL

### CDK Oncoproteins

Cyclin-dependent kinases (CDK) are a family of protein kinases that play an essential role in the regulation of cell cycle progression (Scheicher et al., 2015). The CDK4/6-cyclinD complex is of particular importance due to the crucial role of CDK6 in activating leukemic stem cells (LCSs), which are important for the development of AML and CML (Scheicher et al., 2015). CDK6 is also involved in hematopoietic malignancies such as Mixed-Lineage Leukemia (MLL) related AML (Scheicher et al., 2015) and acute lymphoblastic leukemia (ALL) (Linden et al., 2014). Owning to its critical role in development of hematopoietic malignancies, several small molecule inhibitors targeting the ATP-binding domain of CDK6 have been developed (He et al., 2020). However, the presence of an identical ATP-binding domain on CDK4 (He et al., 2020), thereby preventing CDK6 specific inhibition, along with the scaffolding activity and kinase-independent functions (that promote growth of hematopoietic tumors), make these inhibitors an insufficient treatment (Dominici et al., 2020).

YX-2-107 (Supplementary Table S1) is a PROTAC, designed using palbociclin and CBRN ligands, intended as a possible treatment for acute lymphoblastic leukemia (ALL), involving the Philadelphia chromosome positive (Ph+) ALL (Dominici et al., 2020). The PROTAC was designed to inhibit CDK6 activity as well as preferentially degrade CDK6 over CDK4, two proteins with almost identical amino acid sequences (Dominici et al., 2020). When tested in Ph + ALL cells and xenografts, and mice, YX-2-107 showed promising results, especially regarding selective CDK6 degradation (Dominici et al., 2020). However it requires more development before it can be considered as a possible treatment, especially to improve its half-life and assess its biological impacts (Dominici et al., 2020).

Other CDK6 PROTACs designed for selective degradation of CDK6 over CDK4 using CDK inhibitors and CRBN ligands include BSJ-03-123 (Supplementary Table S1) (against AML) (Brand et al., 2019) and CP-10 (Supplementary Table S1) (most potent degrader from a CDK6 degrader library) which not only degraded mutated CDK6 that underwent overexpression, but also inhibited hematopoietic malignant cell proliferation (Su et al., 2019). Selective CDK degradation was also achieved using VHL (Steinebach et al., 2020) and IAP (Anderson et al., 2020).

### BTK Oncoproteins

Bruton’s tyrosin kinase (BTK) is present in most hematopoietic cells, most notably in B cells (Woyach et al., 2014; Burger, 2019). BTK plays a key role in signal transduction of B cell receptors: the antigen-receptors activate BTK, which in turn transmits or amplifies the signal by stimulating multiple downstream signal cascades, for example nuclear factor-κB (NFκB) (Woyach et al., 2014; Burger, 2019). BTK is not only involved in intracellular signaling, it also involved in the microenvironment around the cell and assists in the growth and survival of tumor cells (Woyach et al., 2014; Burger, 2019). Furthermore, the poor maturation of B cells due to insufficient BCR response in the absence of BTK suggests BTK plays a key role in B cell survival and development as well (Woyach et al., 2014; Burger, 2019). This is further certified by mutations in BTK domains resulting in the development of X-linked agammaglobulinemia, which is characterized by obstructed B cell development (Woyach et al., 2014). BTK is also instrumental in the development of chronic lymphocytic leukemia (CLL), with the current effective treatments including BTK inhibitors such as Ibrutinib (Woyach et al., 2014; Burger et al., 2017; Burger, 2019). Similarly, BTK was also found to be crucial for the progression of AML (S. Huang et al., 2019; S. Li et al., 2021; Rushworth et al., 2014) and Hairy Cell Leukemia (HCL) (Sivina et al., 2012; Rogers et al., 2021).

Interestingly, PROTACs targeting BTK generally use CRBN instead of VHL, due to VHL exhibiting unsatisfactory degrading properties (Buhimschi et al., 2018; Jaime-Figueroa et al., 2020). DD-04-015 (Supplementary Table S2) (resulting from conjugating RN486 (warhead) to CRBN ligand) is a BTK specific PROTAC, designed by comparing its activity against TL12-186, a multiple kinase degrading PROTAC (H.-T. Huang et al., 2018). When MOLM-14 cells were treated with DD-04-015, effective BTK degradation could be observed after just 4 hours (H.-T. Huang et al., 2018). Furthermore, compared to RN486, the PROTAC showed more sustained pharmacodynamics effect (H.-T. Huang et al., 2018).

Certain BTK PROTACs were designed to target the specific C418S mutation in BTK. These included MT-802 (Supplementary Table S2) (Ibrutinib and pomalidomide) which showed full BTK degradation in CLL cells after about 4 hours at 250 nM concentration (Buhimschi et al., 2018). MT-802 also lacks an acrylamide moiety which results in fewer off-target kinase binding, as compared to the inhibitor ibrutinib (Buhimschi et al., 2018). Another such PROTACs is SJF620 (Supplementary Table S2), which showed pharmacokinetically superior results *in vivo*, as compared to MT-802, due to configurational adjustments in the linker and CRBN ligand (Jaime-Figueroa et al., 2020). P13I (Supplementary Table S2) (ibrutinib and pomalidomide) was successful in degrading both mutant BTK and a wild type that is resistant to ibrutinib (Y. Sun et al., 2018). DD-03-007 (Supplementary Table S2) and DD-03-17 (Supplementary Table S2), designed using CGI1746 and thalidomide, showed reduction in BTK levels within 4 hours, even at 100 nM (Dobrovolsky et al., 2019). Noncovalent PROTACs (Buhimschi et al., 2018; Dobrovolsky et al., 2019; Y.; Sun et al., 2018) and covalent PROTACs (irreversible (Xue et al., 2020) and reversible (Guo et al., 2020)) have also been developed against BTK. One such study compared the activity of reversible covalent BTK-degrading PROTACs against their irreversible covalent and noncovalent counterparts (Gabizon et al., 2020). Results showed that while the noncovalent PROTACs was the most effective, the reversible PROTAC (RC-3) still showed high potency and selectivity, thus resulting in fewer off-target reactions (Gabizon et al., 2020).

### BET Oncoproteins

The Bromodomain (BRD) and extraterminal (BET) protein family, known as “epigenetic readers”, consist of BRD2, BRD3, BRD4 and BRDT (specific to germ cells) (Braun & Gardin, 2017; Reyes-Garau et al., 2019). BET proteins are involved in regulating RNA transcription and cell cycle progression by activating RNA polymerase II (Braun & Gardin, 2017; Reyes-Garau et al., 2019). This is accomplished by the binding of the BET proteins to the acetylated histone protein tails, by their two, conserved N-terminal bromodomains (Braun & Gardin, 2017; Reyes-Garau et al., 2019). BRD4 is better understood than the other BET proteins. It is involved in transcriptional regulation by interacting with cyclin T1 and CDK9, and Mediator complex (Reyes-Garau et al., 2019). BRD4 are involved in the progression of AML by activating the genes *c-MYC* and nucleophosmin, through transcription (Braun & Gardin, 2017; Reyes-Garau et al., 2019). Importance of BRD4 in AML can be inferred from the successful use of BET-inhibitors as treatments (Braun & Gardin, 2017; Reyes-Garau et al., 2019; Bill et al., 2021; Lee et al., 2021; Ramsey et al., 2021). In addition, BET-inhibitors have also been used to treat CLL (E. Kim et al., 2020; Ozer et al., 2018; Sundaram et al., 2020).

Remarkably, BET proteins have been a prime target for PROTACs research (Chan et al., 2018; Hines et al., 2019; S. A.; Kim et al., 2019; Ohoka et al., 2019; Zengerle et al., 2015). MZ1 (Supplementary Table S2) (using JQ1 and VHL ligand), has been used in several studies (Zengerle et al., 2015; Gadd et al., 2017; Roy et al., 2019). Zengerle et al. showed its potential for efficient and prolonged intracellular BRD4 degradation, as well as its preferential degradation of BRD4 as compared to BRD2/3 (Zengerle et al., 2015). Gadd et al. used MZ1 to confirm the formation of ternary complexes by PROTACs (Gadd et al., 2017), while Roy et al. used MZ1 to determine ternary complex stability (Roy et al., 2019). Some BET targeting PROTACs were designed by changing the E3 ligase binding moieties. A1874 (Supplementary Table S3), for example, utilized nutlin (for MDM2) and showed 98% degradation, even at nanomolar concentrations (Hines et al., 2019). MDM2 also provided an extra benefit of stabilizing the upregulation of p53 tumor-suppresser gene (Hines et al., 2019). Similarly, TD-428 (Supplementary Table S3) used TD-106 (unique IMiD analog) and displayed degradation of BET protein in 22Rv1 prostate cancer cells (S. A. Kim et al., 2019). Other such PROTACs include CCW 28-3 (Supplementary Table S3) (Ward et al., 2019), using RNF4 E3 ligase, and Kb02 (Supplementary Table S3) (Zhang et al., 2019), an electrophilic PROTAC that uses DCAF16 ligases. Additional BET targeting PROTACs include: macrocyclic PROTAC, a MZ1 with a cyclizing linker, (Testa et al., 2019); BEtd-246 (Supplementary Table S3) against triple-negative breast cancer (Bai et al., 2017); dBET1 (Supplementary Table S3), exhibiting antitumor activity against leukemia (Winter et al., 2015); ARV-825 (Supplementary Table S3) that induces degradation in BL cell lines (Lu et al., 2015); QCA570 (Supplementary Table S3) showing degradation in acute leukemia cell lines at picomolar concentrations (Qin et al., 2018); ARV-771 (Supplementary Table S3) against castration-resistant prostate cancer (CRPC) (Raina et al., 2016); and antibody-drug conjugates involving BRD4 targeting chimeric degraders (Dragovich et al., 2021).

### FLT-3 Oncoproteins

FMS-like tyrosine kinase 3 are membrane bound receptors with two common mutated forms observed in various types of leukemia (Cheng et al., 2018). Internal Tandem Duplication (ITD), present within the juxtamembrane domain of FLT-3 proteins, is most frequently observed in AML (30%) (Cheng et al., 2018). On the other hand, Missense point mutations, found in the tyrosine kinase domain, are more common in ALL (Cheng et al., 2018). Mutated FLT-3 proteins are involved in activating several signaling pathways, for example MAPK/ERK and STAT5, and thus indicate a higher risk of developing leukemia as well as a worse prognosis (Cheng et al., 2018). High levels on FLT-3 can be found in leukemic cells, which in turn is responsible for overexpression of leukemic oncogenes (Cheng et al., 2018). Due to its prominent involvement in leukemia, FLT-3 specific inhibitors, such as quizartinib (Fathi & Chen, 2017), are being used to treat AML, either unaccompanied or in combination with other treatments (Oliva et al., 2021).

TL13-117 and TL13-149 (Supplementary Table S4) were PROTACs designed against AML that target FLT-3 proteins (H.-T. Huang et al., 2018). They were developed by combining quizartinib with a PEG linker and CRBN ligand (H.-T. Huang et al., 2018). However, the results were unexpected, with increased FLT-3 levels in MOLM-14 cells after treatment with PROTACs and reduced degradation by PROTACs as compared to quizartinib (H.-T. Huang et al., 2018). Conversely, PROTACs, which were designed to target ITD mutation in FLT-3 by coupling quizartinib and VHL ligand, showed enhanced selectivity and stimulation of apoptosis in MV4-11 and MOLM-14 cells, as well as in mice (Burslem et al., 2018). Another study supporting a positive outcome of PROTACs targeting FLT-3 proteins combined FF-10101, a novel FLT-3 inhibitor, with CRBN ligand. The study determined reversible covalent PROTACs displayed a much lower half maximal inhibitory concentration than the irreversible covalent (5 folds higher) and reversible noncovalent (34 fold higher) (Guo et al., 2020).

### Other Oncoproteins

While the aforementioned oncoproteins are the most pertinent in various types of leukemia, there are many other oncoproteins that also play a role in the development of leukemia and have thus been targets for PROTACs. These include STAT3 (H. Zhou et al., 2019), Blc-2 (Bond et al., 2020), PLK1 (Mu et al., 2020) and SMARCA2 and SMARCA4 (Farnaby et al., 2019; Kargbo, 2020). As cancer research advances and the exact role of various oncogenes in the development of leukemia becomes more apparent, leukemic cells have become an ideal target for PROTACs. However, more research is still required to design efficient PROTACs that provide better outcomes with lower doses, before it can be used on a large scale.

## Combating Risk of PROTAC Toxicity With Light Control

As developments in PROTACs continue to progress, researcher strive to create PROTACs that are not only essential research entities, but can also be applied clinically. Thus, researchers have turned towards developing light controlled PROTACs to localize its impact, enhance its selectivity (Reynders et al., 2020) and reduce the risk of toxicity due to its catalytic nature (Reynders et al., 2020). PHOTACs (PHOtochemically Targeting Chimeras) are PROTACs that contain azobenzene which allows control of its degrading activity through light waves (Reynders et al., 2020). Reynders et al. developed several PHOTACs, using a variety of protein targets, including oncogenic proteins BRD2/3/4 and FKBP12, and CRBN ligand (Reynders et al., 2020). Of these, PHOTAC-I-3 appeared to be the most efficient (Reynders et al., 2020). RS4; 11 lymphoblastic cells were treated with PHOTACs and then exposed for 72 h to 390-nm light (Reynders et al., 2020). The results were promising, including conformation of no degradation and reduced cytotoxicity in the dark, and the gradual loss of activation by ‘thermal relaxation’ once the PHOTACs are activated (Reynders et al., 2020).

Recently, Jin et al. also utilized azobenzene to design light controlled PROTACs, termed as Azo-PROTAC (Jin et al., 2020). Azo-PROTACs were constructed to target ABL/BCR-ABL (Dasatinib) using lenalidomide to attract CRBN (Jin et al., 2020). Azo-PROTACs were activated using UV-C light and showed promising results in K562 cells (Jin et al., 2020). Their capacity to be controlled by light was confirmed by treating K562 cells with UV-C light and comparing them to treated K562 cells that were not treated (Jin et al., 2020). Those irradiated slowly regained BCR-ABL levels, as opposed to those that were not exposed to light (Jin et al., 2020).

Currently, a number of other researchers have also ventured into developing light-controlled PROTACs to combat its toxicity risks (J. Liu et al., 2021; Pfaff et al., 2019).

## Clinical Trials: PROTACs as an Anticancer Therapy

Thus far, only two PROTACs have been approved for clinical trials. Arvinas, the biotechnology company responsible for both PROTACs, was started in 2013 by Craig Crews from Yale University (Konstantinidou et al., 2019). The first is ARV-110, a small molecule that is orally bioavailable (Neklesa et al., 2018). ARV-110 targets Androgen Receptors involved in prostate cancer (Neklesa et al., 2018). When tested in several cell lines, ARV-110 achieves complete degradation, with 50% degradation at concentrations lower than 1 nM (Neklesa et al., 2018). Similarly, the second PROTAC in clinical trials is ARV-471. This targets the estrogen receptor in breast cancer and also degrades clinically important ESR1 variants (Y537S and D538G) (Flanagan et al., 2019). ARV-471 can achieve 50% protein degradation at concentrations of approximately 2 nM (Flanagan et al., 2019). ARV-471 is also orally available and a dose daily administered in estradiol-dependent MCF7 xenografts showed reduced estrogen receptor levels as well as decreased tumor size (Flanagan et al., 2019).

## Conclusion

Cancer, including leukemia, therapeutics are an ongoing challenge. Currently applied treatments have many drawbacks, primarily drug resistance, ‘undruggable’ proteins and requirement of high doses due to formation of irreversible bonds between the drug and the target. PROTACs provide an appealing solution, with its catalytic mode of action, allosteric binding and capacity to degrade mutant proteins. This review discusses the potential of PROTACs as an anticancer therapy, particularly against leukemia. While results from studies are promising, some aspects of PROTACs need to be developed before they can be used as standardized cancer therapies, particularly designing optimized PROTACs with reduced risk of cytotoxicity.

## References

[B1] An S. Fu L. (2018). Small-molecule PROTACs: An Emerging and Promising Approach for the Development of Targeted Therapy Drugs. EBioMedicine 36, 553–562. 10.1016/j.ebiom.2018.09.005 PubMed Abstract | 10.1016/j.ebiom.2018.09.005 | Google Scholar 30224312 PMC6197674

[B2] Anderson N. A. Cryan J. Ahmed A. Dai H. McGonagle G. A. Rozier C. (2020). Selective CDK6 Degradation Mediated by Cereblon, VHL, and Novel IAP-Recruiting PROTACs. Bioorg. Med. Chem. Lett. 30 (9), 127106. 10.1016/j.bmcl.2020.127106 PubMed Abstract | 10.1016/j.bmcl.2020.127106 | Google Scholar 32184044

[B3] Bai L. Zhou B. Yang C.-Y. Ji J. McEachern D. Przybranowski S. (2017). Targeted Degradation of BET Proteins in Triple-Negative Breast Cancer. Cancer Res. 77 (9), 2476–2487. 10.1158/0008-5472.CAN-16-2622 PubMed Abstract | 10.1158/0008-5472.CAN-16-2622 | Google Scholar 28209615 PMC5413378

[B4] Bill M. Goda C. Pepe F. Ozer H. G. McNeil B. Zhang X. (2021). Targeting BRD4 in Acute Myeloid Leukemia with Partial Tandem Duplication of the MLL Gene. haematol 106 (9), 2527–2532. 10.3324/haematol.2020.271627 10.3324/haematol.2020.271627 | Google Scholar PMC8409020 33979989

[B5] Bond M. J. Chu L. Nalawansha D. A. Li K. Crews C. M. (2020). Targeted Degradation of Oncogenic KRASG12C by VHL-Recruiting PROTACs. ACS Cent. Sci. 6 (8), 1367–1375. 10.1021/acscentsci.0c00411 PubMed Abstract | 10.1021/acscentsci.0c00411 | Google Scholar 32875077 PMC7453568

[B6] Bondeson D. P. Smith B. E. Burslem G. M. Buhimschi A. D. Hines J. Jaime-Figueroa S. (2018). Lessons in PROTAC Design from Selective Degradation with a Promiscuous Warhead. Cell Chem. Biol. 25 (1), 78–87. 10.1016/j.chembiol.2017.09.010 PubMed Abstract | 10.1016/j.chembiol.2017.09.010 | Google Scholar 29129718 PMC5777153

[B7] Bracco E. Shahzad Ali M. Magnati S. Saglio G. (2021). “The Paradigm of Targeting an Oncogenic Tyrosine Kinase: Lesson from BCR-ABL,” in Advances in Precision Medicine Oncology. Editors Arnouk H. Hassan B. A. R.. 10.5772/intechopen.97528 10.5772/intechopen.97528 | Google Scholar

[B8] Brand M. Jiang B. Bauer S. Donovan K. A. Liang Y. Wang E. S. (2019). Homolog-Selective Degradation as a Strategy to Probe the Function of CDK6 in AML. Cell Chem. Biol. 26 (2), 300–306. 10.1016/j.chembiol.2018.11.006 PubMed Abstract | 10.1016/j.chembiol.2018.11.006 | Google Scholar 30595531 PMC6444916

[B9] Braun T. Gardin C. (2017). Investigational BET Bromodomain Protein Inhibitors in Early Stage Clinical Trials for Acute Myelogenous Leukemia (AML). Expert Opin. Investigational Drugs 26 (7), 803–811. 10.1080/13543784.2017.1335711 PubMed Abstract | 10.1080/13543784.2017.1335711 | Google Scholar 28541716

[B10] Bray F. Ferlay J. Soerjomataram I. Siegel R. L. Torre L. A. Jemal A. (2018). Global Cancer Statistics 2018: GLOBOCAN Estimates of Incidence and Mortality Worldwide for 36 Cancers in 185 Countries. CA A Cancer J. Clin. 68 (6), 394–424. 10.3322/caac.21492 PubMed Abstract | 10.3322/caac.21492 | Google Scholar 30207593

[B11] Bricelj A. Steinebach C. Kuchta R. Gütschow M. Sosič I. (2021). E3 Ligase Ligands in Successful PROTACs: An Overview of Syntheses and Linker Attachment Points. Front. Chem. 9. 10.3389/fchem.2021.707317 10.3389/fchem.2021.707317 | Google Scholar PMC8287636 34291038

[B12] Buhimschi A. D. Armstrong H. A. Toure M. Jaime-Figueroa S. Chen T. L. Lehman A. M. (2018). Targeting the C481S Ibrutinib-Resistance Mutation in Bruton's Tyrosine Kinase Using PROTAC-Mediated Degradation. Biochemistry 57 (26), 3564–3575. 10.1021/acs.biochem.8b00391 PubMed Abstract | 10.1021/acs.biochem.8b00391 | Google Scholar 29851337

[B13] Burger J. A. (2019). BTK Inhibitors: Present and Future. Cancer J. 25 (6), 386–393. 10.1097/PPO.0000000000000412 PubMed Abstract | 10.1097/PPO.0000000000000412 | Google Scholar 31764119 PMC7083517

[B14] Burger J. A. Li K. W. Keating M. J. Sivina M. Amer A. M. Garg N. (2017). Leukemia Cell Proliferation and Death in Chronic Lymphocytic Leukemia Patients on Therapy with the BTK Inhibitor Ibrutinib. J. Clin. Investigation 2 (2). 10.1172/jci.insight.89904 PubMed Abstract | 10.1172/jci.insight.89904 | Google Scholar PMC5256142 28138560

[B15] Burslem G. M. Schultz A. R. Bondeson D. P. Eide C. A. Savage Stevens S. L. Druker B. J. (2019). Targeting BCR-ABL1 in Chronic Myeloid Leukemia by PROTAC-Mediated Targeted Protein Degradation. Cancer Res. 79 (18), 4744–4753. 10.1158/0008-5472.CAN-19-1236 PubMed Abstract | 10.1158/0008-5472.CAN-19-1236 | Google Scholar 31311809 PMC6893872

[B16] Burslem G. M. Song J. Chen X. Hines J. Crews C. M. (2018). Enhancing Antiproliferative Activity and Selectivity of a FLT-3 Inhibitor by Proteolysis Targeting Chimera Conversion. J. Am. Chem. Soc. 140 (48), 16428–16432. 10.1021/jacs.8b10320 PubMed Abstract | 10.1021/jacs.8b10320 | Google Scholar 30427680

[B17] Cao S. Ma L. Liu Y. Wei M. Yao Y. Li C. (2021). Proteolysis-Targeting Chimera (PROTAC) Modification of Dovitinib Enhances the Antiproliferative Effect against FLT3-ITD-Positive Acute Myeloid Leukemia Cells. J. Med. Chem. 64 (22), 16497–16511. 10.1021/acs.jmedchem.1c00996 PubMed Abstract | 10.1021/acs.jmedchem.1c00996 | Google Scholar 34694800

[B18] Chan K.-H. Zengerle M. Testa A. Ciulli A. (2018). Impact of Target Warhead and Linkage Vector on Inducing Protein Degradation: Comparison of Bromodomain and Extra-terminal (BET) Degraders Derived from Triazolodiazepine (JQ1) and Tetrahydroquinoline (I-Bet726) BET Inhibitor Scaffolds. J. Med. Chem. 61 (2), 504–513. 10.1021/acs.jmedchem.6b01912 PubMed Abstract | 10.1021/acs.jmedchem.6b01912 | Google Scholar 28595007 PMC5788402

[B19] Chen Y. Jin J. (2020). The Application of Ubiquitin Ligases in the PROTAC Drug Design. Acta Biochimica Biophysica Sinica 52 (7), 776–790. 10.1093/abbs/gmaa053 PubMed Abstract | 10.1093/abbs/gmaa053 | Google Scholar 32506133

[B20] Cheng J. Qu L. Wang J. Cheng L. Wang Y. (2018). High Expression of FLT3 Is a Risk Factor in Leukemia. Mol. Med. Rep. 17 (2), 2885–2892. 10.3892/mmr.2017.8232 PubMed Abstract | 10.3892/mmr.2017.8232 | Google Scholar 29257272 PMC5783504

[B21] Corbin A. S. Agarwal A. Loriaux M. Cortes J. Deininger M. W. Druker B. J. (2011). Human Chronic Myeloid Leukemia Stem Cells Are Insensitive to Imatinib Despite Inhibition of BCR-ABL Activity. J. Clin. Invest. 121 (1), 396–409. 10.1172/JCI35721 PubMed Abstract | 10.1172/JCI35721 | Google Scholar 21157039 PMC3007128

[B22] De Dominici M. Porazzi P. Xiao Y. Chao A. Tang H.-Y. Kumar G. (2020). Selective Inhibition of Ph-Positive ALL Cell Growth through Kinase-dependent and -independent Effects by CDK6-specific PROTACs. Blood 135 (18), 1560–1573. 10.1182/blood.2019003604 PubMed Abstract | 10.1182/blood.2019003604 | Google Scholar 32040545 PMC7193186

[B23] Demizu Y. Shibata N. Hattori T. Ohoka N. Motoi H. Misawa T. (2016). Development of BCR-ABL Degradation Inducers via the Conjugation of an Imatinib Derivative and a cIAP1 Ligand. Bioorg. Med. Chem. Lett. 26 (20), 4865–4869. 10.1016/j.bmcl.2016.09.041 PubMed Abstract | 10.1016/j.bmcl.2016.09.041 | Google Scholar 27666635

[B24] Dobrovolsky D. Wang E. S. Morrow S. Leahy C. Faust T. Nowak R. P. (2019). Bruton Tyrosine Kinase Degradation as a Therapeutic Strategy for Cancer. Blood 133 (9), 952–961. 10.1182/blood-2018-07-862953 PubMed Abstract | 10.1182/blood-2018-07-862953 | Google Scholar 30545835 PMC6396177

[B25] Dong Y. Shi O. Zeng Q. Lu X. Wang W. Li Y. (2020). Leukemia Incidence Trends at the Global, Regional, and National Level between 1990 and 2017. Exp. Hematol. Oncol. 9 (14). 10.1186/s40164-020-00170-6 PubMed Abstract | 10.1186/s40164-020-00170-6 | Google Scholar PMC7304189 32577323

[B26] Dragovich P. S. Pillow T. H. Blake R. A. Sadowsky J. D. Adaligil E. Adhikari P. (2021). Antibody-Mediated Delivery of Chimeric BRD4 Degraders. Part 2: Improvement of *In Vitro* Antiproliferation Activity and *In Vivo* Antitumor Efficacy. J. Med. Chem. 64 (5), 2576–2607. 10.1021/acs.jmedchem.0c01846 PubMed Abstract | 10.1021/acs.jmedchem.0c01846 | Google Scholar 33596073

[B27] Farnaby W. Koegl M. Roy M. J. Whitworth C. Diers E. Trainor N. (2019). BAF Complex Vulnerabilities in Cancer Demonstrated via Structure-Based PROTAC Design. Nat. Chem. Biol. 15 (7), 672–680. 10.1038/s41589-019-0294-6 PubMed Abstract | 10.1038/s41589-019-0294-6 | Google Scholar 31178587 PMC6600871

[B28] Fathi A. T. Chen Y.-B. (2017). The Role of FLT3 Inhibitors in the Treatment of FLT3-Mutated Acute Myeloid Leukemia. Eur. J. Haematol. 98 (4), 330–336. 10.1111/ejh.12841 PubMed Abstract | 10.1111/ejh.12841 | Google Scholar 28000291

[B29] Flanagan J. Qian Y. Gough S. Andreoli M. Bookbinder M. Cadelina G. (2019). ARV-471, an Oral Estrogen Receptor PROTAC Degrader for Breast Cancer . 2018 San Antonio Breast Cancer Symposium. 10.1158/1538-7445.SABCS18-P5-04-18 10.1158/1538-7445.SABCS18-P5-04-18 | Google Scholar

[B30] Gabizon R. Shraga A. Gehrtz P. Livnah E. Shorer Y. Gurwicz N. (2020). Efficient Targeted Degradation via Reversible and Irreversible Covalent PROTACs. J. Am. Chem. Soc. 142 (27), 11734–11742. 10.1021/jacs.9b13907 PubMed Abstract | 10.1021/jacs.9b13907 | Google Scholar 32369353 PMC7349657

[B31] Gadd M. S. Testa A. Lucas X. Chan K.-H. Chen W. Lamont D. J. (2017). Structural Basis of PROTAC Cooperative Recognition for Selective Protein Degradation. Nat. Chem. Biol. 13, 514–521. 10.1038/nchembio.2329 PubMed Abstract | 10.1038/nchembio.2329 | Google Scholar 28288108 PMC5392356

[B32] Guo W.-H. Qi X. Yu X. Liu Y. Chung C.-I. Bai F. (2020). Enhancing Intracellular Accumulation and Target Engagement of PROTACs with Reversible Covalent Chemistry. Nat. Commun. 11. 10.1038/s41467-020-17997-6 PubMed Abstract | 10.1038/s41467-020-17997-6 | Google Scholar PMC7450057 32848159

[B33] He Y. Khan S. Huo Z. Lv D. Zhang X. Liu X. (2020). Proteolysis Targeting Chimeras (PROTACs) Are Emerging Therapeutics for Hematologic Malignancies. J. Hematol. Oncol. 13 (103). 10.1186/s13045-020-00924-z PubMed Abstract | 10.1186/s13045-020-00924-z | Google Scholar PMC7384229 32718354

[B34] Hines J. Lartigue S. Dong H. Qian Y. Crews C. M. (2019). MDM2-Recruiting PROTAC Offers Superior, Synergistic Antiproliferative Activity via Simultaneous Degradation of BRD4 and Stabilization of P53. Cancer Res. 79 (1), 251–262. 10.1158/0008-5472.CAN-18-2918 PubMed Abstract | 10.1158/0008-5472.CAN-18-2918 | Google Scholar 30385614 PMC6318015

[B35] Hu B. Zhou Y. Sun D. Yang Y. Liu Y. Li X. (2020). PROTACs: New Method to Degrade Transcription Regulating Proteins. Eur. J. Med. Chem. 207, 112698. 10.1016/j.ejmech.2020.112698 PubMed Abstract | 10.1016/j.ejmech.2020.112698 | Google Scholar 32858471

[B36] Huang H.-T. Dobrovolsky D. Paulk J. Yang G. Weisberg E. L. Doctor Z. M. (2018). A Chemoproteomic Approach to Query the Degradable Kinome Using a Multi-Kinase Degrader. Cell Chem. Biol. 25 (1), 88–99. 10.1016/j.chembiol.2017.10.005 PubMed Abstract | 10.1016/j.chembiol.2017.10.005 | Google Scholar 29129717 PMC6427047

[B37] Huang S. Pan J. Jin J. Li C. Li X. Huang J. (2019). Abivertinib, a Novel BTK Inhibitor: Anti-leukemia Effects and Synergistic Efficacy with Homoharringtonine in Acute Myeloid Leukemia. Cancer Lett. 461, 132–143. 10.1016/j.canlet.2019.07.008 PubMed Abstract | 10.1016/j.canlet.2019.07.008 | Google Scholar 31310800

[B38] Huang T.-T. Wang X. Qiang S.-J. Zhao Z.-N. Wu Z.-X. Ashby C. R. (2021). The Discovery of Novel BCR-ABL Tyrosine Kinase Inhibitors Using a Pharmacophore Modeling and Virtual Screening Approach. Front. Cell Dev. Biol. 9. 10.3389/fcell.2021.649434 PubMed Abstract | 10.3389/fcell.2021.649434 | Google Scholar PMC7969810 33748144

[B39] Jaime-Figueroa S. Buhimschi A. D. Toure M. Hines J. Crews C. M. (2020). Design, Synthesis and Biological Evaluation of Proteolysis Targeting Chimeras (PROTACs) as a BTK Degraders with Improved Pharmacokinetic Properties. Bioorg. Med. Chem. Lett. 30 (3), 126877. 10.1016/j.bmcl.2019.126877 PubMed Abstract | 10.1016/j.bmcl.2019.126877 | Google Scholar 31879210 PMC7318425

[B40] Jin Y.-H. Lu M.-C. Wang Y. Shan W.-X. Wang X.-Y. You Q.-D. (2020). Azo-PROTAC: Novel Light-Controlled Small-Molecule Tool for Protein Knockdown. J. Med. Chem. 63 (9), 4644–4654. 10.1021/acs.jmedchem.9b02058 PubMed Abstract | 10.1021/acs.jmedchem.9b02058 | Google Scholar 32153174

[B41] Kargbo R. B. (2020). SMARCA2/4 PROTAC for Targeted Protein Degradation and Cancer Therapy. ACS Med. Chem. Lett. 11 (10), 1797–1798. 10.1021/acsmedchemlett.0c00347 PubMed Abstract | 10.1021/acsmedchemlett.0c00347 | Google Scholar 33062156 PMC7549100

[B42] Khan S. He Y. Zhang X. Yuan Y. Pu S. Kong Q. (2020). PROteolysis TArgeting Chimeras (PROTACs) as Emerging Anticancer Therapeutics. Oncogene 39 (26), 4909–4924. 10.1038/s41388-020-1336-y PubMed Abstract | 10.1038/s41388-020-1336-y | Google Scholar 32475992 PMC7319888

[B43] Kim E. ten Hacken E. Sivina M. Clarke A. Thompson P. A. Jain N. (2020). The BET Inhibitor GS-5829 Targets Chronic Lymphocytic Leukemia Cells and Their Supportive Microenvironment. Leukemia 34, 1588–1598. 10.1038/s41375-019-0682-7 PubMed Abstract | 10.1038/s41375-019-0682-7 | Google Scholar 31862959 PMC7272263

[B44] Kim S. A. Go A. Jo S.-H. Park S. J. Jeon Y. U. Kim J. E. (2019). A Novel Cereblon Modulator for Targeted Protein Degradation. Eur. J. Med. Chem. 166, 65–74. 10.1016/j.ejmech.2019.01.023 PubMed Abstract | 10.1016/j.ejmech.2019.01.023 | Google Scholar 30684871

[B45] Konstantinidou M. Li J. Zhang B. Wang Z. Shaabani S. Ter Brake F. (2019). PROTACs- a Game-Changing Technology. Expert Opin. Drug Discov. 14 (12), 1255–1268. 10.1080/17460441.2019.1659242 PubMed Abstract | 10.1080/17460441.2019.1659242 | Google Scholar 31538491 PMC7008130

[B46] Lai A. C. Toure M. Hellerschmied D. Salami J. Jaime-Figueroa S. Ko E. (2015). Modular PROTAC Design for the Degradation of Oncogenic BCR-ABL. Angew. Chem. Int. Ed. 55 (2), 807–810. 10.1002/anie.201507634 PubMed Abstract | 10.1002/anie.201507634 | Google Scholar PMC4733637 26593377

[B47] Lee L. Hizukuri Y. Severson P. Powell B. Zhang C. Ma Y. (2021). A Novel Combination Regimen of BET and FLT3 Inhibition for FLT3-ITD Acute Myeloid Leukemia. haematol 106 (4), 1022–1033. 10.3324/haematol.2020.247346 10.3324/haematol.2020.247346 | Google Scholar PMC8017818 33504139

[B48] Li S. Wu B. Zheng X. Wang C. Zhao J. Sun H. (2021). Synthesis and Biological Activity of Imidazole Group-Substituted Arylaminopyrimidines (IAAPs) as Potent BTK Inhibitors against B-Cell Lymphoma and AML. Bioorg. Chem. 106, 104385. 10.1016/j.bioorg.2020.104385 PubMed Abstract | 10.1016/j.bioorg.2020.104385 | Google Scholar 33272709

[B49] Li X. Song Y. (2020). Proteolysis-targeting Chimera (PROTAC) for Targeted Protein Degradation and Cancer Therapy. J. Hematol. Oncol. 13 (50). 10.1186/s13045-020-00885-3 PubMed Abstract | 10.1186/s13045-020-00885-3 | Google Scholar PMC7218526 32404196

[B50] Liu H H. Ding X. Liu L. Mi Q. Zhao Q. Shao Y. (2021). Discovery of Novel BCR-ABL PROTACs Based on the Cereblon E3 Ligase Design, Synthesis, and Biological Evaluation. Eur. J. Med. Chem. 223 (5), 113645. 10.1016/j.ejmech.2021.113645 PubMed Abstract | 10.1016/j.ejmech.2021.113645 | Google Scholar 34217059

[B51] Liu J J. Peng Y. Wei W. (2021). Light-Controllable PROTACs for Temporospatial Control of Protein Degradation. Front. Cell Dev. Biol. 9. 10.3389/fcell.2021.678077 PubMed Abstract | 10.3389/fcell.2021.678077 | Google Scholar PMC8326567 34350175

[B52] Liu J. Ma J. Liu Y. Xia J. Li Y. Wang Z. P. (2020). PROTACs: A Novel Strategy for Cancer Therapy. Seminars Cancer Biol. 67, 171–179. 10.1016/j.semcancer.2020.02.006 PubMed Abstract | 10.1016/j.semcancer.2020.02.006 | Google Scholar 32058059

[B53] Lu J. Qian Y. Altieri M. Dong H. Wang J. Raina K. (2015). Hijacking the E3 Ubiquitin Ligase Cereblon to Efficiently Target BRD4. Chem. Biol. 22 (6), 755–763. 10.1016/j.chembiol.2015.05.009 PubMed Abstract | 10.1016/j.chembiol.2015.05.009 | Google Scholar 26051217 PMC4475452

[B54] Mahon F.-X. Réa D. Guilhot J. Guilhot F. Huguet F. Nicolini F. (2010). Discontinuation of Imatinib in Patients with Chronic Myeloid Leukaemia Who Have Maintained Complete Molecular Remission for at Least 2 years: the Prospective, Multicentre Stop Imatinib (STIM) Trial. Lancet Oncol. 11 (11), 1029–1035. 10.1016/S1470-2045(10)70233-3 PubMed Abstract | 10.1016/S1470-2045(10)70233-3 | Google Scholar 20965785

[B55] Maniaci C. Hughes S. J. Testa A. Chen W. Lamont D. J. Rocha S. (2017). Homo-PROTACs: Bivalent Small-Molecule Dimerizers of the VHL E3 Ubiquitin Ligase to Induce Self-Degradation. Nat. Commun. 8 (830). 10.1038/s41467-017-00954-1 PubMed Abstract | 10.1038/s41467-017-00954-1 | Google Scholar PMC5635026 29018234

[B56] Miranda-Filho A. Piñeros M. Ferlay J. Soerjomataram I. Monnereau A. Bray F. (2018). Epidemiological Patterns of Leukaemia in 184 Countries: a Population-Based Study. Lancet Haematol. 5 (1), e14–24. 10.1016/S2352-3026(17)30232-6 PubMed Abstract | 10.1016/S2352-3026(17)30232-6 | Google Scholar 29304322

[B57] Mu X. Bai L. Xu Y. Wang J. Lu H. (2020). Protein Targeting Chimeric Molecules Specific for Dual Bromodomain 4 (BRD4) and Polo-like Kinase 1 (PLK1) Proteins in Acute Myeloid Leukemia Cells. Biochem. Biophysical Res. Commun. 521 (4), 833–839. 10.1016/j.bbrc.2019.11.007 PubMed Abstract | 10.1016/j.bbrc.2019.11.007 | Google Scholar 31708096

[B58] Neklesa T. Snyder L. B. Willard R. R. Vitale N. Raina K. Pizzano J. (2018). ARV-110: An Androgen Receptor PROTAC Degrader for Prostate Cancer. AACR Annu. Meet. 10.1158/1538-7445.AM2018-5236 10.1158/1538-7445.AM2018-5236 | Google Scholar

[B59] Ohoka N. Tsuji G. Shoda T. Fujisato T. Kurihara M. Demizu Y. (2019). Development of Small Molecule Chimeras that Recruit AhR E3 Ligase to Target Proteins. ACS Chem. Biol. 14 (12), 2822–2832. 10.1021/acschembio.9b00704 PubMed Abstract | 10.1021/acschembio.9b00704 | Google Scholar 31580635

[B60] Oliva J. Villanueva L. Ochiai J. Niihara Y. (2021). New Anti-cancer Drug Compounds to Treat FLT-3 Mutated Leukemia. Blood 138, 4349. 10.1182/blood-2021-153894 10.1182/blood-2021-153894 | Google Scholar

[B61] Ozer H. G. El-Gamal D. Powell B. Hing Z. A. Blachly J. S. Harrington B. (2018). BRD4 Profiling Identifies Critical Chronic Lymphocytic Leukemia Oncogenic Circuits and Reveals Sensitivity to PLX51107, a Novel Structurally Distinct BET Inhibitor. Cancer Discov. 8 (4), 458–477. 10.1158/2159-8290.CD-17-0902 PubMed Abstract | 10.1158/2159-8290.CD-17-0902 | Google Scholar 29386193 PMC5882533

[B62] Paiva S.-L. Crews C. M. (2019). Targeted Protein Degradation: Elements of PROTAC Design. Curr. Opin. Chem. Biol. 50, 111–119. 10.1016/j.cbpa.2019.02.022 PubMed Abstract | 10.1016/j.cbpa.2019.02.022 | Google Scholar 31004963 PMC6930012

[B63] Pfaff P. Samarasinghe K. T. G. Crews C. M. Carreira E. M. (2019). Reversible Spatiotemporal Control of Induced Protein Degradation by Bistable PhotoPROTACs. ACS Cent. Sci. 5 (10), 1682–1690. 10.1021/acscentsci.9b00713 PubMed Abstract | 10.1021/acscentsci.9b00713 | Google Scholar 31660436 PMC6813558

[B64] Pophali P. A. Patnaik M. M. (2016). The Role of New Tyrosine Kinase Inhibitors in Chronic Myeloid Leukemia. Cancer J. 22 (1), 40–50. 10.1097/PPO.0000000000000165 PubMed Abstract | 10.1097/PPO.0000000000000165 | Google Scholar 26841016 PMC4742366

[B65] Qi S.-M. Dong J. Xu Z.-Y. Cheng X.-D. Zhang W.-D. Qin J.-J. (2021). PROTAC: An Effective Targeted Protein Degradation Strategy for Cancer Therapy. Front. Pharmacol. 12. 10.3389/fphar.2021.692574 10.3389/fphar.2021.692574 | Google Scholar PMC8138175 34025443

[B66] Qin C. Hu Y. Zhou B. Fernandez-Salas E. Yang C.-Y. Liu L. (2018). Discovery of QCA570 as an Exceptionally Potent and Efficacious Proteolysis Targeting Chimera (PROTAC) Degrader of the Bromodomain and Extra-terminal (BET) Proteins Capable of Inducing Complete and Durable Tumor Regression. J. Med. Chem. 61 (15), 6685–6704. 10.1021/acs.jmedchem.8b00506 PubMed Abstract | 10.1021/acs.jmedchem.8b00506 | Google Scholar 30019901 PMC6545111

[B67] Raina K. Lu J. Qian Y. Altieri M. Gordon D. Rossi A. M. K. (2016). PROTAC-induced BET Protein Degradation as a Therapy for Castration-Resistant Prostate Cancer. Proc. Natl. Acad. Sci. U.S.A. 113 (26), 7124–7129. 10.1073/pnas.1521738113 PubMed Abstract | 10.1073/pnas.1521738113 | Google Scholar 27274052 PMC4932933

[B68] Ramsey H. E. Greenwood D. Zhang S. Childress M. Arrate M. P. Gorska A. E. (2021). BET Inhibition Enhances the Antileukemic Activity of Low-Dose Venetoclax in Acute Myeloid Leukemia. Clin. Cancer Res. 27, 598–607. 10.1158/1078-0432.CCR-20-1346 PubMed Abstract | 10.1158/1078-0432.CCR-20-1346 | Google Scholar 33148670

[B69] Reyes-Garau D. Ribeiro M. L. Roué G. (2019). Pharmacological Targeting of BET Bromodomain Proteins in Acute Myeloid Leukemia and Malignant Lymphomas: From Molecular Characterization to Clinical Applications. Cancers 11 (10), 1483. 10.3390/cancers11101483 PubMed Abstract | 10.3390/cancers11101483 | Google Scholar PMC6826405 31581671

[B70] Reynders M. Matsuura B. S. Bérouti M. Simoneschi D. Marzio A. Pagano M. (2020). PHOTACs Enable Optical Control of Protein Degradation. Sci. Adv. 6 (8), eaay5064. 10.1126/sciadv.aay5064 PubMed Abstract | 10.1126/sciadv.aay5064 | Google Scholar 32128406 PMC7034999

[B71] Rogers K. A. Andritsos L. A. Wei L. McLaughlin E. M. Ruppert A. S. Anghelina M. (2021). Phase 2 Study of Ibrutinib in Classic and Variant Hairy Cell Leukemia. Blood 137 (25), 3473–3483. 10.1182/blood.2020009688 PubMed Abstract | 10.1182/blood.2020009688 | Google Scholar 33754642 PMC8225920

[B72] Roy M. J. Winkler S. Hughes S. J. Whitworth C. Galant M. Farnaby W. (2019). SPR-measured Dissociation Kinetics of PROTAC Ternary Complexes Influence Target Degradation Rate. ACS Chem. Biol. 14 (3), 361–368. 10.1021/acschembio.9b00092 PubMed Abstract | 10.1021/acschembio.9b00092 | Google Scholar 30721025 PMC6423499

[B73] Ru Y. Wang Q. Liu X. Zhang M. Zhong D. Ye M. (2016). The Chimeric Ubiquitin Ligase SH2-U-Box Inhibits the Growth of Imatinib-Sensitive and Resistant CML by Targeting the Native and T315I-Mutant BCR-ABL. Sci. Rep. 6 (11), 1–13. 10.1038/srep28352 PubMed Abstract | 10.1038/srep28352 | Google Scholar 27329306 PMC4916441

[B74] Rushworth S. A. Murray M. Y. Zaitseva L. Bowles K. M. MacEwan D. J. (2014). Identification of Bruton's Tyrosine Kinase as a Therapeutic Target in Acute Myeloid Leukemia. Blood 123 (8), 1229–1238. 10.1182/blood-2013-06-511154 PubMed Abstract | 10.1182/blood-2013-06-511154 | Google Scholar 24307721

[B75] Sakamoto K. M. Kim K. B. Kumagai A. Mercurio F. Crews C. M. Deshaies R. J. (2001). Protacs: Chimeric Molecules that Target Proteins to the Skp1-Cullin-F Box Complex for Ubiquitination and Degradation. Proc. Natl. Acad. Sci. U.S.A. 98 (15), 8554–8559. 10.1073/pnas.141230798 PubMed Abstract | 10.1073/pnas.141230798 | Google Scholar 11438690 PMC37474

[B76] Sakamoto K. M. (2010). Protacs for Treatment of Cancer. Pediatr. Res. 67 (5), 505–508. 10.1203/PDR.0b013e3181d35017 PubMed Abstract | 10.1203/PDR.0b013e3181d35017 | Google Scholar 20075761 PMC2881331

[B77] Scheicher R. Hoelbl-Kovacic A. Bellutti F. Tigan A.-S. Prchal-Murphy M. Heller G. (2015). CDK6 as a Key Regulator of Hematopoietic and Leukemic Stem Cell Activation. Blood 125 (1), 90–101. 10.1182/blood-2014-06-584417 PubMed Abstract | 10.1182/blood-2014-06-584417 | Google Scholar 25342715 PMC4281832

[B78] Shibata N. Miyamoto N. Nagai K. Shimokawa K. Sameshima T. Ohoka N. (2017). Development of Protein Degradation Inducers of Oncogenic BCR ˗ ABL Protein by Conjugation of ABL Kinase Inhibitors and IAP Ligands. Cancer Sci. 108 (8), 1657–1666. 10.1111/cas.13284 PubMed Abstract | 10.1111/cas.13284 | Google Scholar 28556300 PMC5543464

[B79] Shimokawa K. Shibata N. Sameshima T. Miyamoto N. Ujikawa O. Nara H. (2017). Targeting the Allosteric Site of Oncoprotein BCR-ABL as an Alternative Strategy for Effective Target Protein Degradation. ACS Med. Chem. Lett. 8 (10), 1042–1047. 10.1021/acsmedchemlett.7b00247 PubMed Abstract | 10.1021/acsmedchemlett.7b00247 | Google Scholar 29057048 PMC5641955

[B80] Sivina M. Kreitman R. J. Arons E. Buggy J. J. Ravandi F. Burger J. A. (2012). Bruton's Tyrosine Kinase (BTK) Inhibitor Ibrutinib (PCI-32765) Blocks Hairy Cell Leukemia (HCL) Survival, Proliferation, and BCR Signaling: A New Therapeutic Approach for HCL. Blood 120 (21), 1802. 10.1182/blood.V120.21.1802.1802 10.1182/blood.V120.21.1802.1802 | Google Scholar

[B81] Smith B. E. Wang S. L. Jaime-Figueroa S. Harbin A. Wang J. Hamman B. D. (2019). Differential PROTAC Substrate Specificity Dictated by Orientation of Recruited E3 Ligase. Nat. Commun. 10 (131). 10.1038/s41467-018-08027-7 PubMed Abstract | 10.1038/s41467-018-08027-7 | Google Scholar PMC6328587 30631068

[B82] Steinebach C. Ng Y. L. D. Sosič I. Lee C.-S. Chen S. Lindner S. (2020). Systematic Exploration of Different E3 Ubiquitin Ligases: an Approach towards Potent and Selective CDK6 Degraders. Chem. Sci. 11, 3474–3486. 10.1039/D0SC00167H PubMed Abstract | 10.1039/D0SC00167H | Google Scholar 33133483 PMC7552917

[B83] Su S. Yang Z. Gao H. Yang H. Zhu S. An Z. (2019). Potent and Preferential Degradation of CDK6 via Proteolysis Targeting Chimera Degraders. J. Med. Chem. 62 (15), 7575–7582. 10.1021/acs.jmedchem.9b00871 PubMed Abstract | 10.1021/acs.jmedchem.9b00871 | Google Scholar 31330105 PMC6790125

[B84] Sun X. Gao H. Yang Y. He M. Wu Y. Song Y. (2019). PROTACs: Great Opportunities for Academia and Industry. Sig Transduct. Target Ther. 4 (64). 10.1038/s41392-019-0101-6 PubMed Abstract | 10.1038/s41392-019-0101-6 | Google Scholar PMC6927964 31885879

[B85] Sun Y. Zhao X. Ding N. Gao H. Wu Y. Yang Y. (2018). PROTAC-induced BTK Degradation as a Novel Therapy for Mutated BTK C481S Induced Ibrutinib-Resistant B-Cell Malignancies. Cell Res. 28, 779–781. 10.1038/s41422-018-0055-1 PubMed Abstract | 10.1038/s41422-018-0055-1 | Google Scholar 29875397 PMC6028582

[B86] Sundaram S. Mavis C. Gu J. J. Torka P. Hernandez-Ilizaliturri F. J. (2020). BRD4 Inhibitors Enhance the Anti-tumor Activity of Targeted Therapy in Chronic Lymphocytic Leukemia. Blood 136, 37. 10.1182/blood-2020-143237 10.1182/blood-2020-143237 | Google Scholar

[B87] Sung H. Ferlay J. Siegel R. L. Laversanne M. Soerjomataram I. Jemal A. (2021). Global Cancer Statistics 2020: GLOBOCAN Estimates of Incidence and Mortality Worldwide for 36 Cancers in 185 Countries. CA A Cancer J. Clin. 71 (3), 209–249. 10.3322/caac.21660 10.3322/caac.21660 | Google Scholar 33538338

[B88] Testa A. Hughes S. J. Lucas X. Wright J. E. Ciulli A. (2019). Structure˗Based Design of a Macrocyclic PROTAC. Angew. Chem. Int. Ed. 59 (4), 1727–1734. 10.1002/anie.201914396 PubMed Abstract | 10.1002/anie.201914396 | Google Scholar PMC7004083 31746102

[B89] Tong B. Spradlin J. N. Novaes L. F. T. Zhang E. Hu X. Moeller M. (2020). A Nimbolide-Based Kinase Degrader Preferentially Degrades Oncogenic BCR-ABL. ACS Chem. Biol. 15 (7), 1788–1794. 10.1021/acschembio.0c00348 PubMed Abstract | 10.1021/acschembio.0c00348 | Google Scholar 32568522 PMC7891886

[B90] Troup R. I. Fallan C. Baud M. G. J. (2020). Current Strategies for the Design of PROTAC Linkers: a Critical Review. Explor. Target. Anti-Tumor Ther. 1, 273–312. 10.37349/etat.2020.00018 10.37349/etat.2020.00018 | Google Scholar PMC9400730 36046485

[B91] Tsukahara F. Maru Y. (2010). Bag1 Directly Routes Immature BCR-ABL for Proteasomal Degradation. Blood 116 (18), 3582–3592. 10.1182/BLOOD-2009-10-249623 PubMed Abstract | 10.1182/BLOOD-2009-10-249623 | Google Scholar 20675402

[B92] Van der Linden M. Willekes M. van Roon E. Seslija L. Schneider P. Pieters R. (2014). MLL Fusion-Driven Activation ofCDK6potentiates Proliferation inMLL-Rearranged Infant ALL. Cell Cycle 13 (5), 834–844. 10.4161/cc.27757 PubMed Abstract | 10.4161/cc.27757 | Google Scholar 24736461 PMC3979919

[B93] Ward C. C. Kleinman J. I. Brittain S. M. Lee P. S. Chung C. Y. S. Kim K. (2019). Covalent Ligand Screening Uncovers a RNF4 E3 Ligase Recruiter for Targeted Protein Degradation Applications. ACS Chem. Biol. 14 (11), 2430–2440. 10.1021/acschembio.8b01083 PubMed Abstract | 10.1021/acschembio.8b01083 | Google Scholar 31059647 PMC7422721

[B94] Weng G. Shen C. Cao D. Gao J. Dong X. He Q. (2021). PROTAC-DB: an Online Database of PROTACs. Nucleic Acids Research2 49, D1381–D1387. 10.1093/nar/gkaa807 10.1093/nar/gkaa807 | Google Scholar PMC7778940 33010159

[B95] Winter G. E. Buckley D. L. Paulk J. Roberts J. M. Souza A. Dhe-Paganon S. (2015). Phthalimide Conjugation as a Strategy for *In Vivo* Target Protein Degradation. Science 348 (6241), 1376–1381. 10.1126/science.aab1433 PubMed Abstract | 10.1126/science.aab1433 | Google Scholar 25999370 PMC4937790

[B96] Woyach J. A. Bojnik E. Ruppert A. S. Stefanovski M. R. Goettl V. M. Smucker K. A. (2014). Bruton's Tyrosine Kinase (BTK) Function Is Important to the Development and Expansion of Chronic Lymphocytic Leukemia (CLL). Blood 123 (8), 1207–1213. 10.1182/blood-2013-07-515361 PubMed Abstract | 10.1182/blood-2013-07-515361 | Google Scholar 24311722 PMC3931190

[B97] Xue G. Chen J. Liu L. Zhou D. Zuo Y. Fu T. (2020). Protein Degradation through Covalent Inhibitor-Based PROTACs. Chem. Commun. 56, 1521–1524. 10.1039/C9CC08238G 10.1039/C9CC08238G | Google Scholar 31922153

[B98] Yang Y. Gao H. Sun X. Sun Y. Qiu Y. Weng Q. (2020). Global PROTAC Toolbox for Degrading BCR-ABL Overcomes Drug-Resistant Mutants and Adverse Effects. J. Med. Chem. 63 (15), 8567–8583. 10.1021/acs.jmedchem.0c00967 PubMed Abstract | 10.1021/acs.jmedchem.0c00967 | Google Scholar 32657579

[B99] Yin L. Hu Q. (2020). Chimera Induced Protein Degradation: PROTACs and beyond. Eur. J. Med. Chem. 206, 112494. 10.1016/j.ejmech.2020.112494 PubMed Abstract | 10.1016/j.ejmech.2020.112494 | Google Scholar 32890974

[B100] Zengerle M. Chan K.-H. Ciulli A. (2015). Selective Small Molecule Induced Degradation of the BET Bromodomain Protein BRD4. ACS Chem. Biol. 10 (8), 1770–1777. 10.1021/acschembio.5b00216 PubMed Abstract | 10.1021/acschembio.5b00216 | Google Scholar 26035625 PMC4548256

[B101] Zhang X. Crowley V. M. Wucherpfennig T. G. Dix M. M. Cravatt B. F. (2019). Electrophilic PROTACs that Degrade Nuclear Proteins by Engaging DCAF16. Nat. Chem. Biol. 15, 737–746. 10.1038/s41589-019-0279-5 PubMed Abstract | 10.1038/s41589-019-0279-5 | Google Scholar 31209349 PMC6592777

[B102] Zhao Q. Ren C. Liu L. Chen J. Shao Y. Sun N. (2019). Discovery of SIAIS178 as an Effective BCR-ABL Degrader by Recruiting Von Hippel-Lindau (VHL) E3 Ubiquitin Ligase. J. Med. Chem. 62 (20), 9281–9298. 10.1021/acs.jmedchem.9b01264 PubMed Abstract | 10.1021/acs.jmedchem.9b01264 | Google Scholar 31539241

[B103] Zhou H. Bai L. Xu R. Zhao Y. Chen J. McEachern D. (2019). Structure-Based Discovery of SD-36 as a Potent, Selective, and Efficacious PROTAC Degrader of STAT3 Protein. J. Med. Chem. 62 (24), 11280–11300. 10.1021/acs.jmedchem.9b01530 PubMed Abstract | 10.1021/acs.jmedchem.9b01530 | Google Scholar 31747516 PMC8848307

[B104] Zhou X. Dong R. Zhang J.-Y. Zheng X. Sun L.-P. (2020). PROTAC: A Promising Technology for Cancer Treatment. Eur. J. Med. Chem. 203, 112539. 10.1016/j.ejmech.2020.112539 PubMed Abstract | 10.1016/j.ejmech.2020.112539 | Google Scholar 32698111

