# Supplementary material for: PROTACs: The Future of Leukemia Therapeutics
Source: Front Cell Dev Biol. 2022 Sep 2;10:851087. doi: 10.3389/fcell.2022.851087 (PMC9479449; doi:10.3389/fcell.2022.851087)

| TABLE 3 \| PROTACs designed against CDK4/6 (Structures taken from PROTAC-DB (<http://cadd.zju.edu.cn/protacdb/>))   \| PROTAC \| Warhead \| Linker \| E3 Ligand \| \| --- \| --- \| --- \| --- \| \| 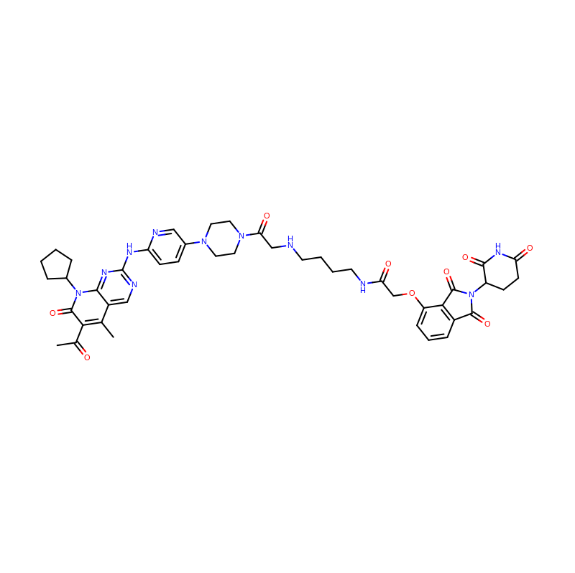  YX-2-107 \| 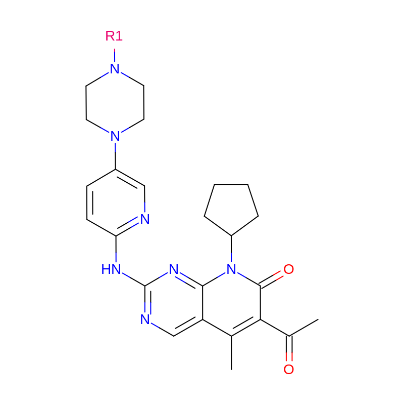 \| 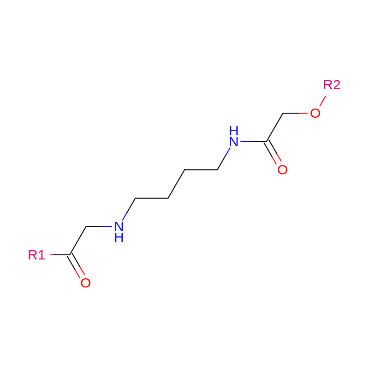 \| 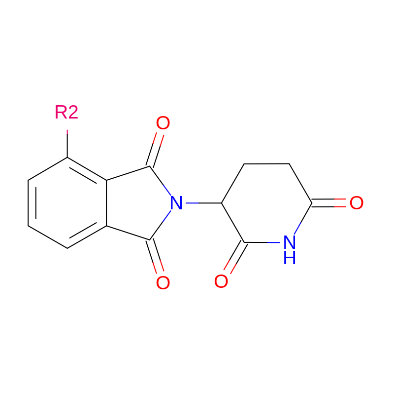  E3 ligase: CRBN \| \| 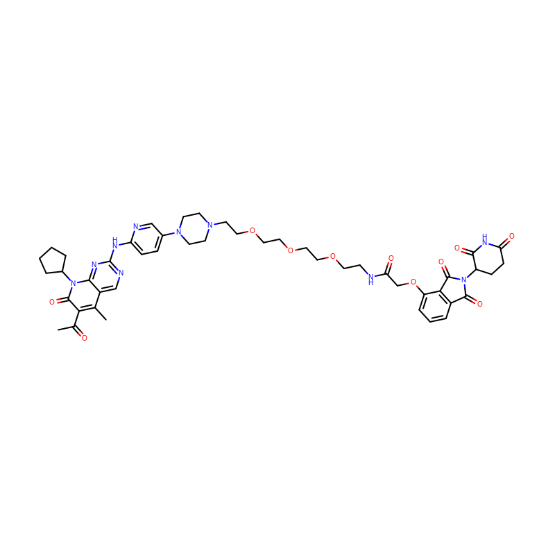  BSJ-03-123 \| 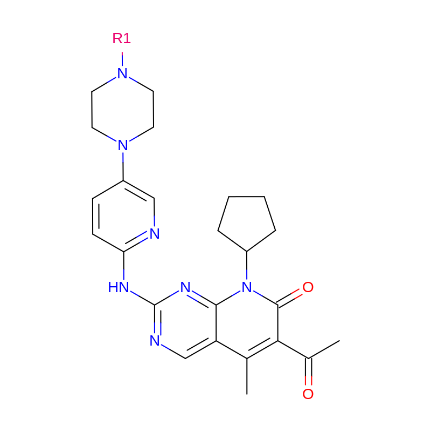 \| 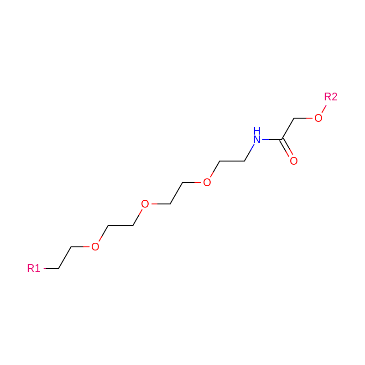 \| 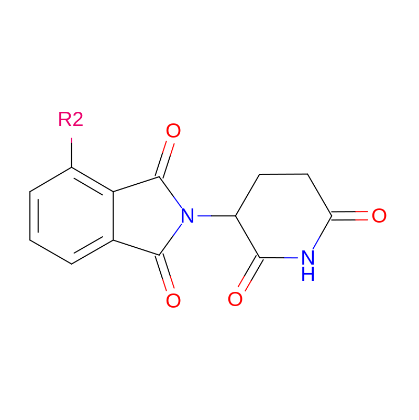  E3 ligase: CRBN \| |
| --- | --- | --- | --- | --- | --- | --- | --- | --- | --- | --- | --- | --- |

| TABLE 3 \| (*Continued*) PROTACs designed against CDK4/6 (Structures taken from PROTAC-DB (<http://cadd.zju.edu.cn/protacdb/>))   \| PROTAC \| Warhead \| Linker \| E3 Ligand \| \| --- \| --- \| --- \| --- \| \| 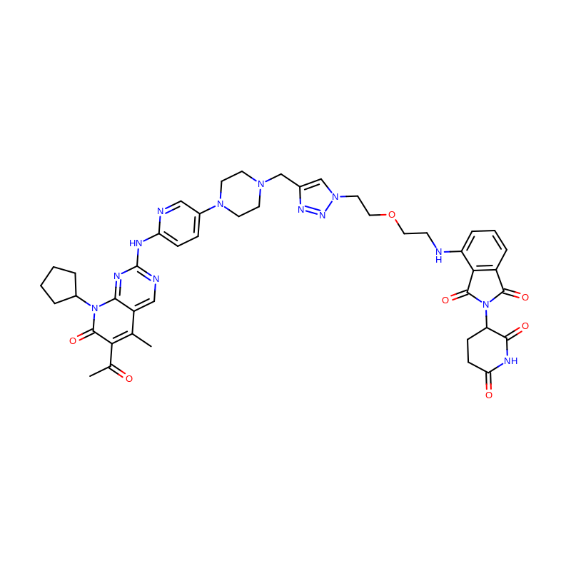  CP-10 \| 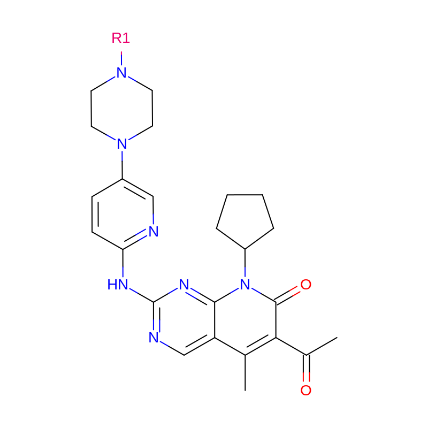 \| 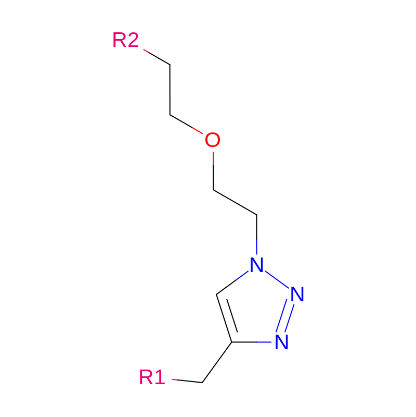 \| 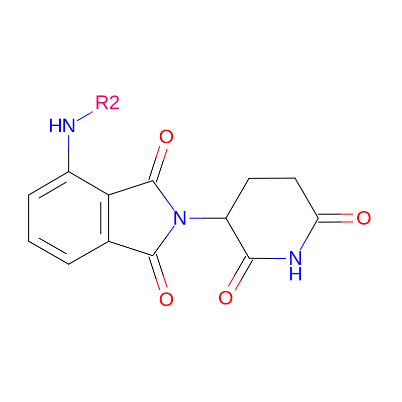  E3 ligase: CRBN \| |
| --- | --- | --- | --- | --- | --- | --- | --- | --- |

| TABLE 4 \| PROTACs designed against BTK (Structures taken from PROTAC-DB (<http://cadd.zju.edu.cn/protacdb/>))   \| PROTAC \| Warhead \| Linker \| E3 Ligand \| \| --- \| --- \| --- \| --- \| \| 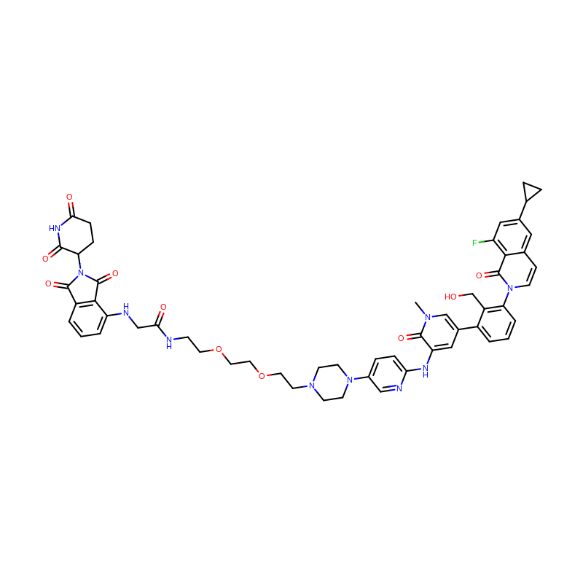  DD-04-015 \| 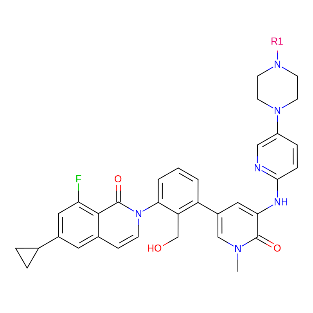 \| 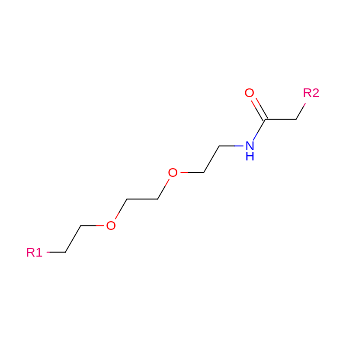 \| 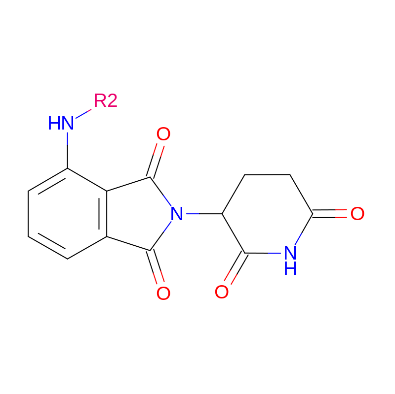  E3 ligase: CRBN \| \| 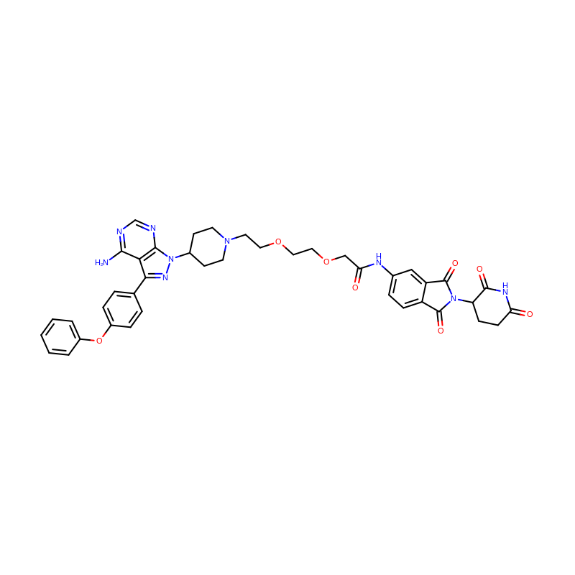  MT-802 \| 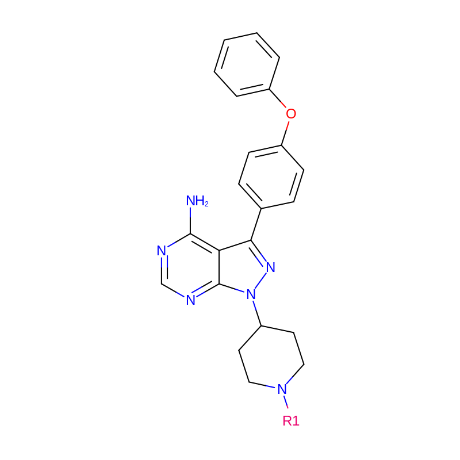 \| 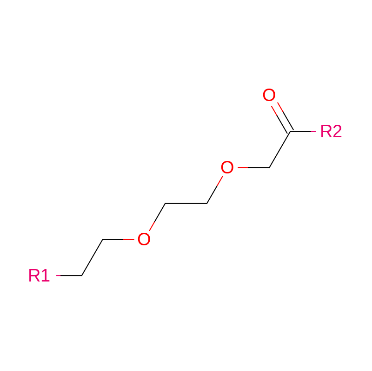 \| 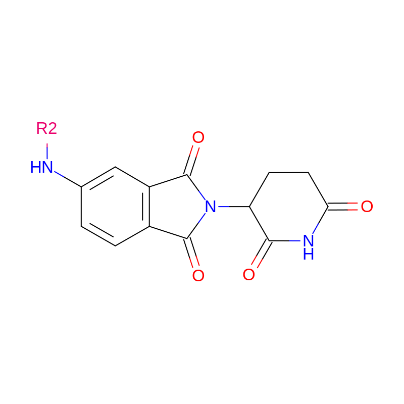  E3 ligase: CRBN \| |
| --- | --- | --- | --- | --- | --- | --- | --- | --- | --- | --- | --- | --- |

TABLE 4 | (Continued) PROTACs designed against BTK (Structures taken from PROTAC-DB (<http://cadd.zju.edu.cn/protacdb/>))

| PROTAC | Warhead | Linker | E3 Ligand |
| --- | --- | --- | --- |
| 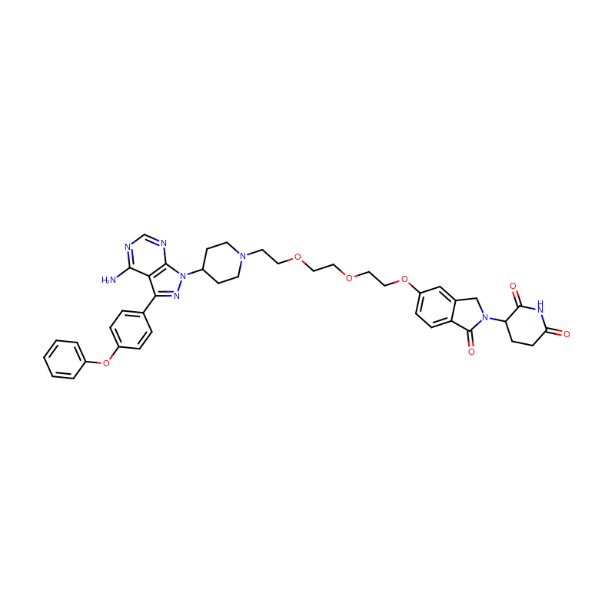  SJF620 | 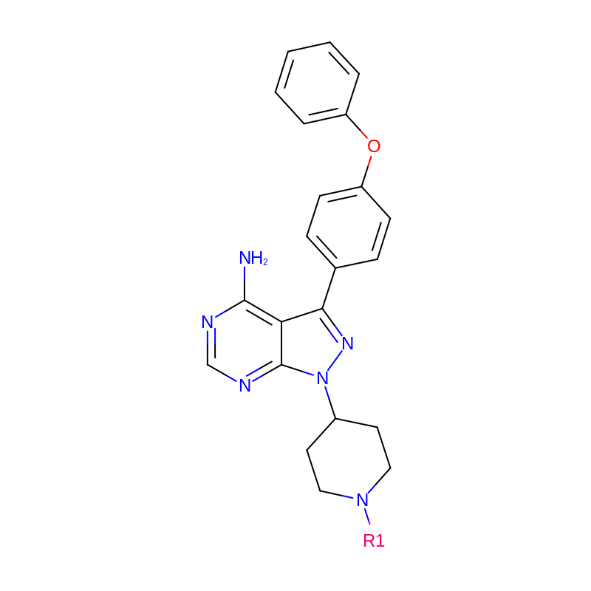 | 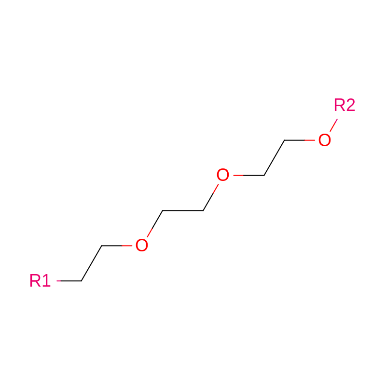 | 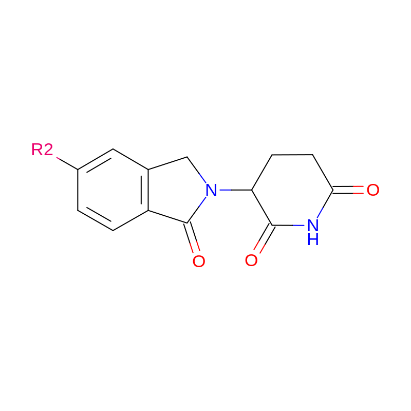  E3 ligase: CRBN |
| 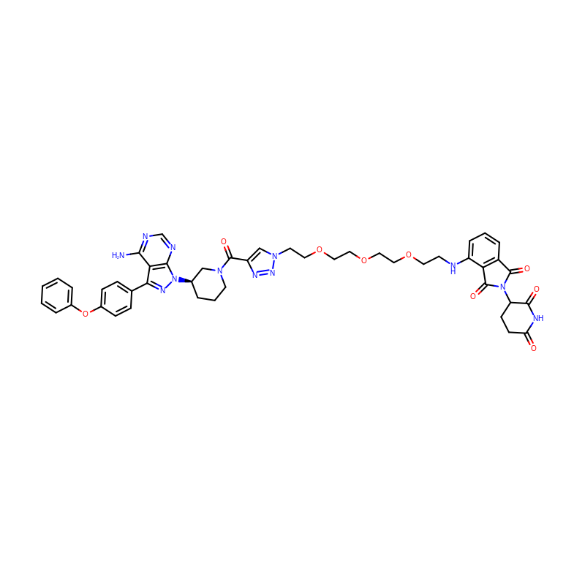  P13I | 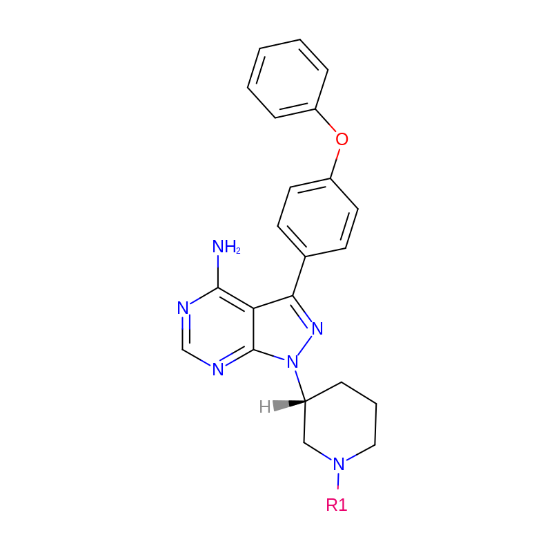 | 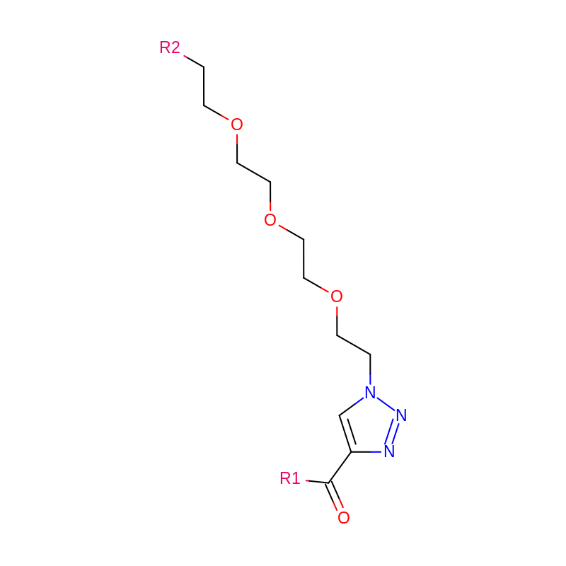 | 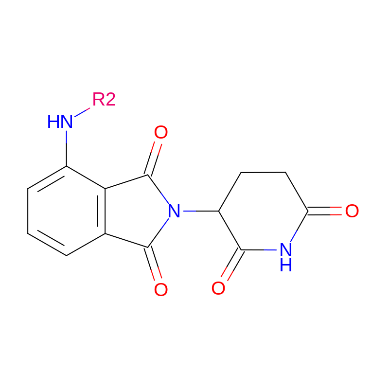  E3 ligase: CRBN |

| TABLE 4 \| (Continued) PROTACs designed against BTK (Structures taken from PROTAC-DB (<http://cadd.zju.edu.cn/protacdb/>))   \| PROTAC \| Warhead \| Linker \| E3 Ligand \| \| --- \| --- \| --- \| --- \| \| 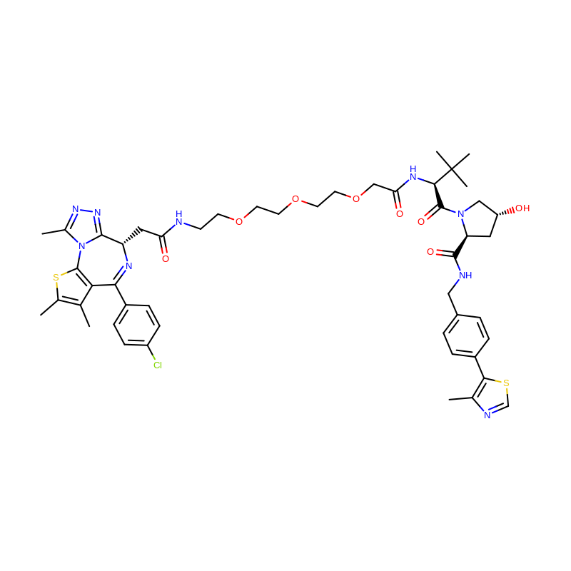  MZ1 \| 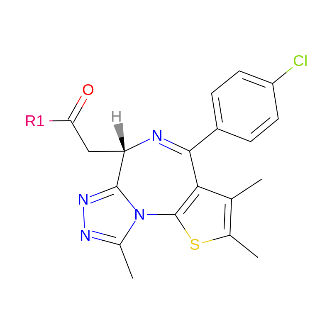 \| 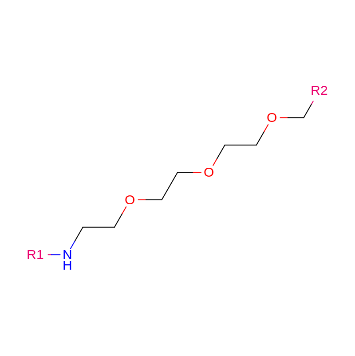 \| 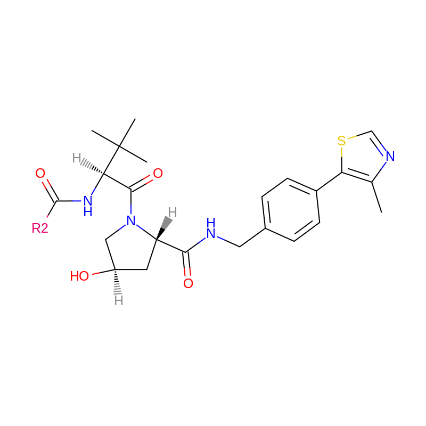  E3 ligase: VHL \| |
| --- | --- | --- | --- | --- | --- | --- | --- | --- |


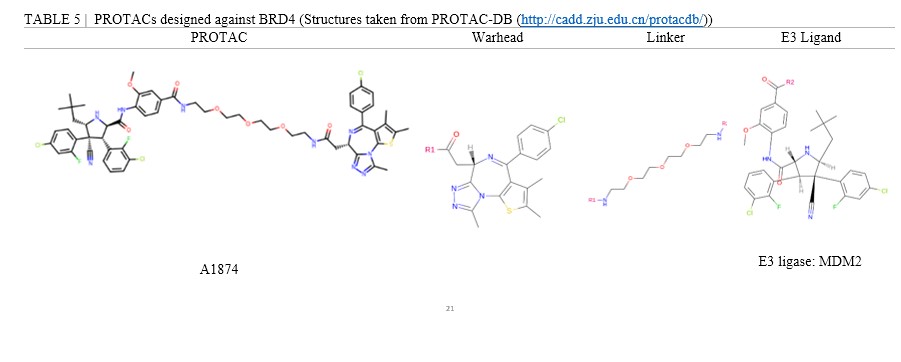


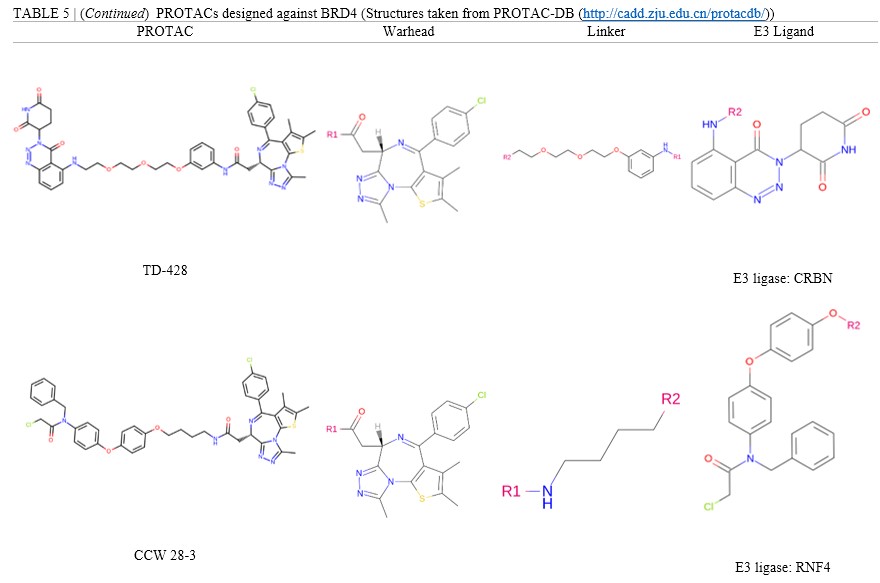

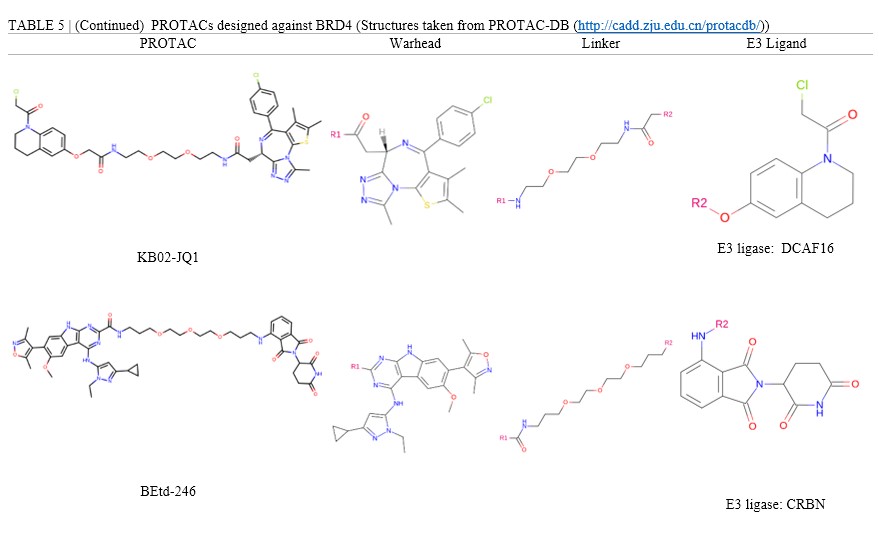


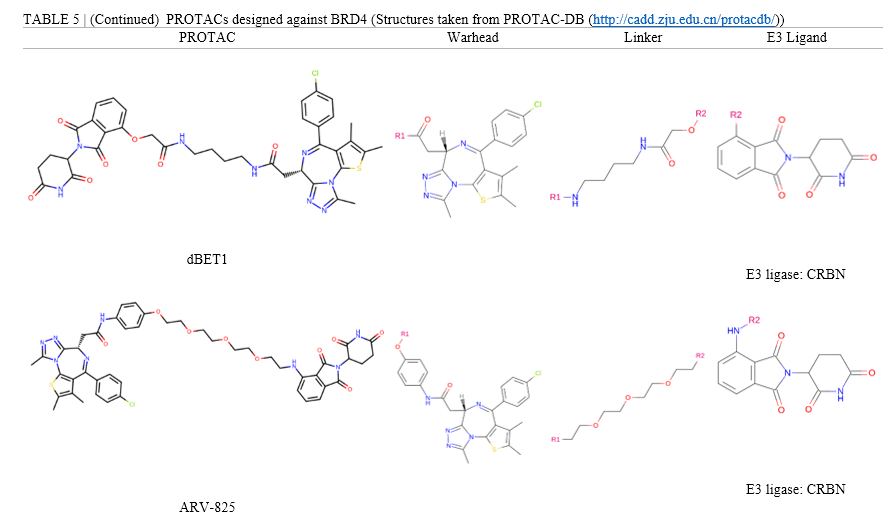


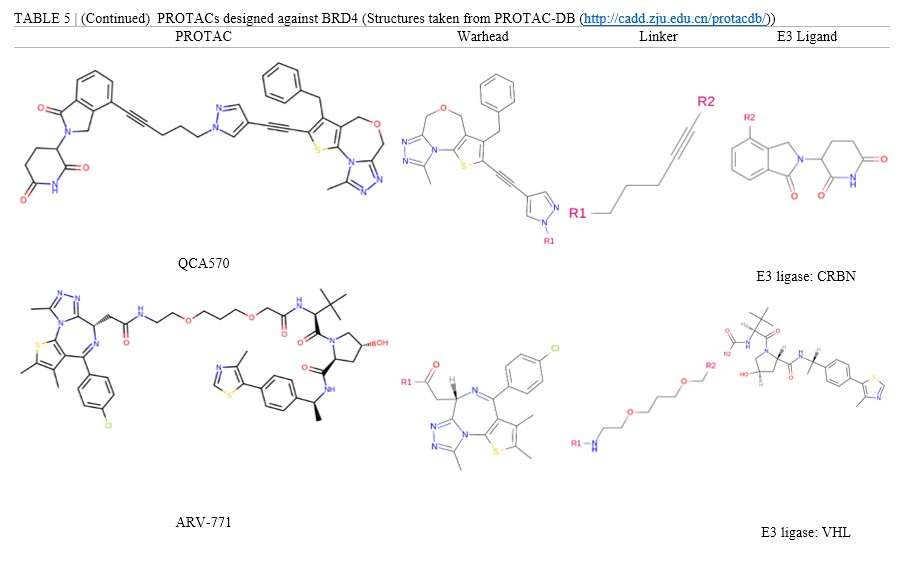


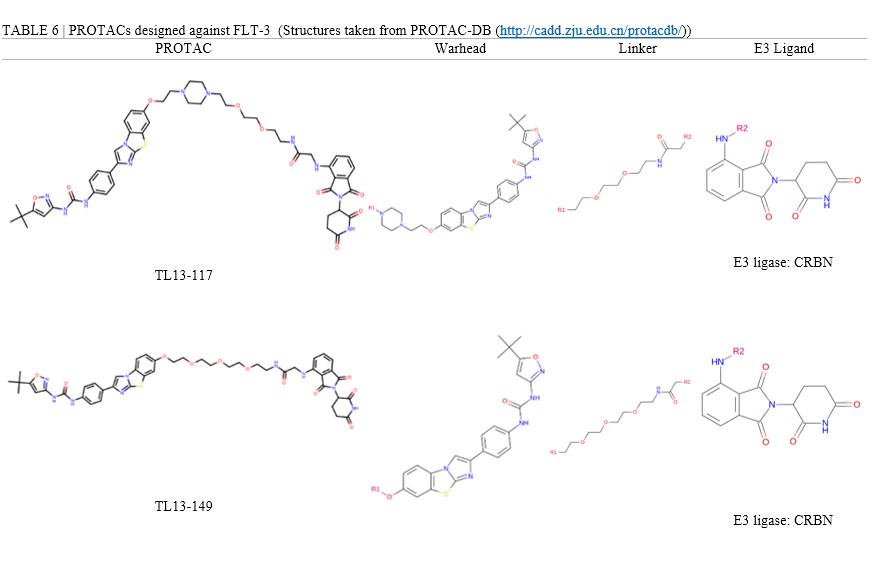

Supplement: Supplementary file 1 [file DataSheet1.docx]
